# Supplementary material for: Novel estimates reveal subnational heterogeneities in disease-relevant contact patterns in the United States
Source: PLoS Comput Biol. 2022 Dec 2;18(12):e1010742. doi: 10.1371/journal.pcbi.1010742 (PMC9749998; doi:10.1371/journal.pcbi.1010742)
Supplement: S1 Appendix — Fig A. The R0 value implied by contact patterns in each state. Fig B. State-specific MRP estimate of total contact (combined household and non-household contacts). Fig C. Illustration of partial pooling. Fig D. Monthly standard deviation of contact intensity across states. Fig E. Model input and output for state of California. Fig F. State-level change in relative contact intensity index over time from a model without mobility data predictors. Fig G—Fig T. Estimated State-level Contact Matrices by month. Table A. Model selection metrics. Table B. Model performance with and without the Oxford Stringency Score. (PDF) [file pcbi.1010742.s001.pdf]

Supporting Information

Novel Estimates Reveal Subnational Heterogeneities in Disease-Relevant Contact Patterns in the United States

Casey F. Breen [*] Ayesha S. Mahmud [†] Dennis M. Feehan [‡]

November 24, 2022

[*]Corresponding author. Department of Demography, University of California, Berkeley. casey-breen@berkeley.edu.

[†]Department of Demography, University of California, Berkeley.

[‡]Department of Demography, University of California, Berkeley.

Contents

1 BICS Contact Survey **3**
1.1 Contact-level Weights . . . 3
1.2 Youngest Age Group . . . 4

2 Methodological Details **4**
2.1 Reciprocity Constraints . . . 4
2.2 Poststratrification . . . 5
2.3 Model Selection . . . 6
2.4 Summarizing contact intensity . . . 7
2.5 Alternative Contact Intensity Measures . . . 8

3 Additional Analyses **12**
3.1 Internal validation check . . . 12
3.2 Variation in Contact Intensity . . . 12
3.3 Mobility data illustration for CA . . . 14
3.4 Model without mobility predictors . . . 14
3.5 Model Code . . . 17
3.6 Contact Matrices by month . . . 18

1 BICS Contact Survey

1.1 Contact-level Weights

Respondents were asked to report the age and sex of all household contact members and to provide detailed information on up to three non-household contacts, including contact age, sex, contact location, and their relation to the contact. In all waves, some respondents reported more than three non-household contacts, but these respondents were prompted to report only on the first three contacts that came to mind. To account for this, within-respondent weights were constructed to provide detailed information on all contacts. Each of the three initial contacts receives the following weight a_i:

$$a_i = \frac{\text{Total number of contacts}}{\text{Total number of contacts for which detailed data was collected}}. \tag{1}$$

To illustrate, if respondent i reports 12 contacts and provides detailed information on 3 contacts, each of the 3 original contacts are assigned a weight $a_i = 4$. Intuitively, the within-respondent weights represent the total number of contacts represented by each of the three contacts who were reported about in detail. This approach assumes that the first three contacts who come to mind are a randomly selected sample of all contacts. For a more detailed explanation, see Feehan and Cobb [8]. We round each respondent's weighted number of contacts in each age category to the closest integer using stochastic rounding:

$$\text{Round}(contacts) = \begin{cases} \lfloor contacts \rfloor, & \text{with probability } 1 - (contacts - \lfloor contacts \rfloor) \\ \lfloor contacts \rfloor + 1, & \text{with probability } (contacts - \lfloor contacts \rfloor) \end{cases}$$

For example, if the weighted number of alters in a certain age category was 5.3 alters, it would be rounded to 5 with probability 0.7 and to 6 with probability 0.3. Stochastic rounding preserves the expected value of the number of weighted contacts, which might not be the case for conventional rounding.

1.2 Youngest Age Group

For the youngest age group $[0-18)$, for which there are no survey respondents, we use the principle of reciprocity to impute the average daily number of connections [7]. Specifically, we assume that the number of contacts for the youngest age group is:

$$\hat{c}_{1j} = \frac{c_{j1} N_j}{N_1} \tag{2}$$

where c_{ij} is the unadjusted, average daily contacts respondents in age group i reported to their contacts in age group j, and N_j is the population size of group j. Intuitively, we are using reciprocity principles to infer the number of connections from the $[0-18)$ group to other groups using reports from other groups to the $[0-18)$ group. However, we cannot use this method to infer the average daily number of contacts the $[0-18)$ group has to other members of the $[0-18)$ group. To do this, we calculate the leading eigenvalue of each of our contact matrices and compare it to the leading eigenvalue of the contact matrix from the UK POLYMOD. We then scale the number of contacts in the UK POLYMOD $[0-18)$ by the ratio of the leading eigenvalues; we use this imputed value for the average daily contacts from members of the $[0-18)$ group to contacts in the $[0-18)$ group.

While we must make several assumptions to estimate contact matrices for the (0-18] age class, these assumptions do not affect our estimates of contact patterns among adults.

2 Methodological Details

2.1 Reciprocity Constraints

We aggregated our poststratified estimates of contact rates by state and time into crude contact matrices and then adjusted these crude contact matrices to enforce reciprocity, an important feature of contact matrices. To simply motivate why contact matrices should be reciprocal: if person A had conversational contact with person B, person B must have also had conversational contact with person A. Therefore, a contact matrix should have the same total number of contacts from age-group i to age-group j as from age group j to age group

i. In practice, estimated contact patterns from survey data (and poststratified estimates) are not necessarily reciprocal. To ensure reciprocity [13], we use the following formula to estimate adjusted mean contacts $\hat{c}_{ij}$, the average number of contacts that a member of group i has with people in group j:

$$\hat{c}_{ij} = \frac{1}{2N_i} \times (c_{ij}N_i + c_{ji}N_j) \tag{3}$$

where

- N_i = size of age-group i from ACS
- N_j = size of age-group j from ACS
- c_{ij} = estimated average number of contacts that a member of group i has with people in group j
- c_{ji} = estimated average number of contacts that a member of group j has with people in group i

2.2 Poststratrification

Survey-based estimates are commonly adjusted using poststratification, a technique that incorporates auxiliary information about the population of interest to improve estimates from a sample. The traditional poststratification approach first assigns respondents to mutually exclusive "cells" – groups cross-classified by key covariates. For example, one cell might be women aged 25-35 living in New York. Next, the cell-level estimates are generated by taking the mean sample response within each cell. Finally, the cell-level estimates are aggregated by weighting each cell relative to its proportion in the population.

This classic poststratification approach works well when the assumptions it relies upon are satisfied. In particular, classic poststratification assumes that each person within a cell has the same chance of being sampled. This assumption is generally violated in practice unless cells have been very finely partitioned. However, once cells have been finely partitioned, the number of interviews per cell typically becomes very small, resulting in unstable cell-level averages. Thus, under classic poststratification there is a tension between how finely cells are partitioned and how stable cell-level average estimates are.

	Mobility	Time Trend	ELPD (Diff)	SE Diff
Model 3	Yes	national cubic + state-specific cubic random effect	0.0	0.0
Model 2	No	national cubic + state-specific cubic random effect	-1.2	6.89
Model 1	No	state-specific cubic random effect	-60.9	17.9

Table A: Model selection metrics. A larger ELPD denotes a more predictive model. This table shows that Model 3 is the best model, as Model 3 has the lowest ELPD.

Hierarchical models like the one proposed here have the potential to help resolve this tension. The idea is to overcome the instability of estimates from very finely partitioned cells by estimating cell-level averages from a hierarchical model that allows for "random" or "modeled" effects for some predictors. Random effects allow for partial pooling towards the group mean, with greater pooling for less-populated cells [6]: when little to no data are available for a cell, estimates are based on data from similar cells; when lots of data are available for a cell, estimates are closer to the original survey responses.

2.3 Model Selection

We fit a series of models with different levels of complexity to identify a model that is as flexible as possible without overfitting. To perform model selection, we use Pareto-smoothed importance sampling leave-one-out cross validation (PSIS LOO-CV) [14]. Conventional LOO-CV requires omitting a single observation and refitting the model. However, for complex models or larger datasets, this can be computationally expensive (e.g., for our model, LOO-CV would require repeating estimation 8,920 times). PSIS LOO-CV is a computationally efficient method for estimating the pointwise out-of-sample predictive accuracy of a model using the Pareto-smoothed importance sampling. PSIS LOO-CV calculates importance weights which come from evaluating the ratio of a set of draws from the full posterior distribution to a set of draws from the leave-one-out posterior distribution. PSIS LOO-CV then fits a Pareto distribution to the upper tail of importance weights to smooth out extreme values. The results are then combined into a single measure, the expected log pointwise predictive density (ELPD). The ELPD is a summary of out-of-sample predictive accuracy using posterior simulations, and can be used both to assess the (out-of-sample) predictive accuracy

Model	ELPD (Diff)	SE Diff
Model 3	0.0	0.0
Model 3 + Oxford Stringency Index	-8.4	6.7

Table B: Incorporating the Oxford Stringency Score into our best-performing model (model 3) decreased our models performance, as measured by the expected log predictive density (ELPD). A larger ELPD denotes a more predictive model.

of the model and for model comparison. Table A summarizes the ELPD predictive density for four different model fits. Based on these results, we chose Model 3 for our analysis: it had the best performance as measured by its LOO-CV ELPD.

Fits shown here were obtained using the `brms` package [1]. Models were run with 1,000 warm-up iterations and 1,000 sampling iterations. Each model was run on four different sampling chains; the R-Hat statistics were between 1 and 1.01, suggesting that the chains had mixed successfully.

Table B shows that incorporating the Oxford Stringency Score [9]—an NPI that measures the stringency of "lock-down" policies that primarily restrict people's social behavior—as a covariate into our model led to a slight decrease in model performance. Understanding why incorporating NPIs into our model did not improve its predictive accuracy is a potential avenue for future research.

2.4 Summarizing contact intensity

While a contact matrix contains valuable detail about age-specific transmission rates, a single summary measure of total level of social contact can facilitate comparisons across time and place. We use the leading eigenvalue of the contact matrix C as a summary measure of the overall levels of contact implied by a contact matrix.

To motivate this choice, we briefly review the link between the contact matrix C and the next generation matrix N, which is useful in modeling the early stages of a disease outbreak; see Diekmann, Heesterbeek and Metz [5] and Diekmann, Heesterbeek and Britton [4] for a full account. The next generation matrix $N = (n_{ij})$ is an $A \times A$ matrix that maps a vector with counts of infected individuals in each age group at generation g, $\boldsymbol{\phi}_g$, to a vector of counts of infected individuals in each age group in the subsequent generation, $\boldsymbol{\phi}_{g+1} = N\boldsymbol{\phi}_g$.

The dominant eigenvalue of the next generation matrix, $\rho(N)$, is the basic reproductive number, R_0 [5]. R_0 is a critical quantity in infectious disease epidemiology: it summarizes the number of secondary infections expected to result from one infected individual in an entirely susceptible population.

The social contact hypothesis says that the next generation matrix is proportional to the contact matrix, i.e., $N = \alpha C$ where $\alpha > 0$ is a proportionality constant that depends on the pathogen and other factors, assuming that there is no age-specific heterogeneity in susceptibility and transmissibility [16, 10, 4]. Under this hypothesis, the dominant eigenvalue of the social contact matrix is $\rho(C) = \rho(\alpha N) = \alpha R_0$. Thus, the dominant eigenvalue of a social contact matrix can be a useful summary of the aggregate level of contact.

The social contact hypothesis can also help compare contact matrices across place or time. Given two contact matrices C_1 and C_2, there will be R_0 values associated with each one, say R_0^1 and R_0^2. Assume that the pathogen of interest is the same and that factors other than contact are held constant, so that the proportionality constant α is the same for both matrices. Then under the social contact hypothesis, $\frac{\rho(C_1)}{\rho(C_2)} = \frac{\alpha R_0^1}{\alpha R_0^2} = \frac{R_0^1}{R_0^2}$. Thus, if the dominant eigenvalue of C_2 is twice the dominant eigenvalue of C_1, then the R_0 value associated with C_2 is twice as big as the R_0 value associated with C_1. Under the social contact hypothesis, this ratio can be identified even if the proportionality constant α is not known.

2.5 Alternative Contact Intensity Measures

In the main body of our paper, we quantify contact rates using the relative contact intensity. Here, we present two alternative measures of contact intensity: (1) the average number of total daily contacts and (2) a measure of R_0 for a respiratory pathogen such as COVID-19 (with age-specific differences in the probability of transmission given contact with an infectious individual). We calculate R_0 using a deterministic, age-stratified compartmental model that has been used previously to study COVID-19 dynamics [3]. The model assumes that all people are initially susceptible, and then can become exposed through a contact with an infected person. After exposure, there is a latent period before individuals become infected either clinically or sub-clinically. Sub-clinical infections are assumed to be less infectious compared with clinical infections. After the infectious state, individuals then

move to the recovered state. To calculate R_0, we take the absolute value of the leading eigenvalue of the next-generation matrix (NGM), which is defined as [3]:

$$NGM = D_u C D_y D_x \tag{4}$$

where

- D_u is a diagonal matrix with elements u_i, the probability of transmission given contact with an infected person for an individual in age-class i (i.e. the relative susceptibility of individuals in age-class i)
- C is the state-specific age-structured contact matrix
- D_y is a diagonal matrix with elements y corresponding to the mean infectious period
- D_x is a diagonal matrix with elements $\rho_j + (1 - \alpha)\rho_j$; ρ_j is the probability of having clinical symptoms in age-class j and α is the relative infectiousness of sub-clinical cases

The probability of transmission given contact with an infected person in a given age-class (u_i) and the probability of having clinical symptoms per age group (ρ_j) were drawn from the literature [3]. We assume a relative infectiousness of clinical vs. subclinical individuals (α) of 0.5 [12] and a mean infectious period of 6 days [11]. We re-scale these u_i values such that a model fit to a baseline contact matrix from UK participants in the POLYMOD study [13, 7, 2] produces the R_0 value of 2.5 [15]. We then fit this model with the rescaled u_i values on all estimated contact matrix; estimates are shown in Figure A.

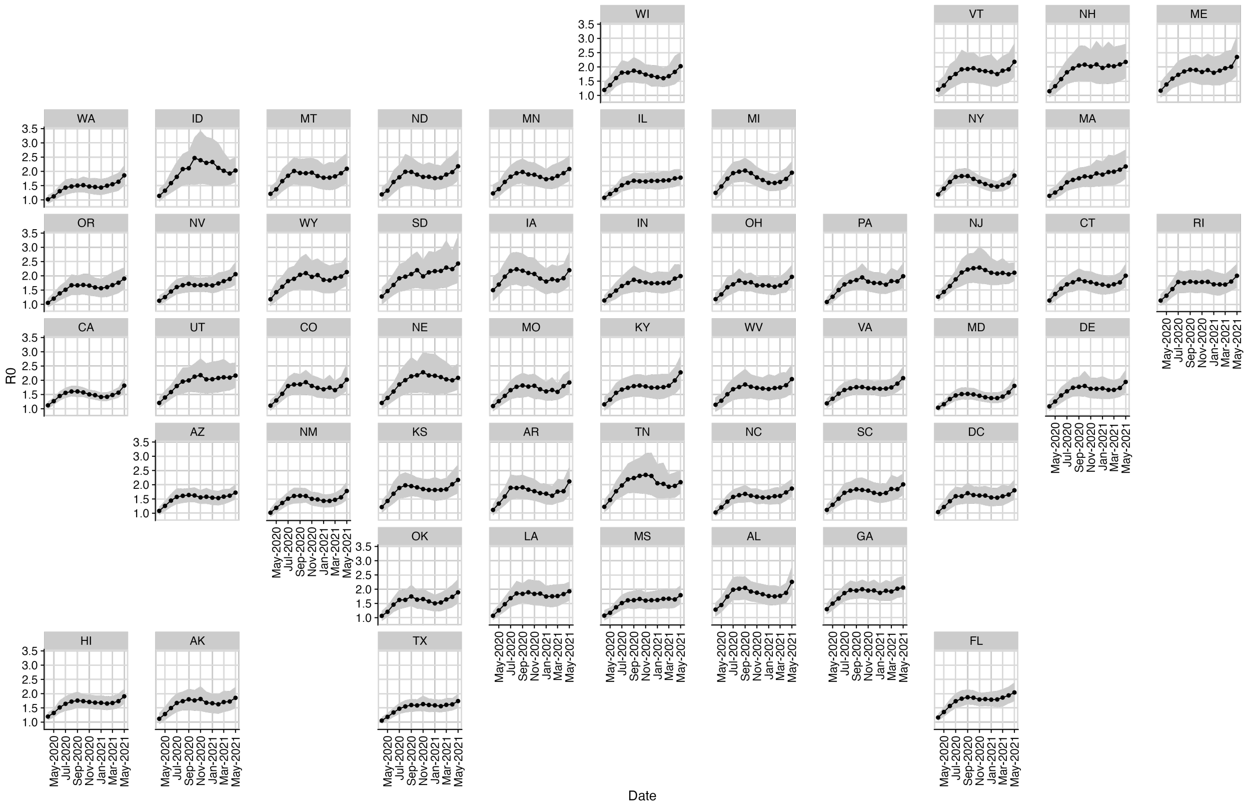

Figure A: The R_0 value implied by contact patterns in each state. R_0 was estimated using an age-stratified deterministic compartmental model.

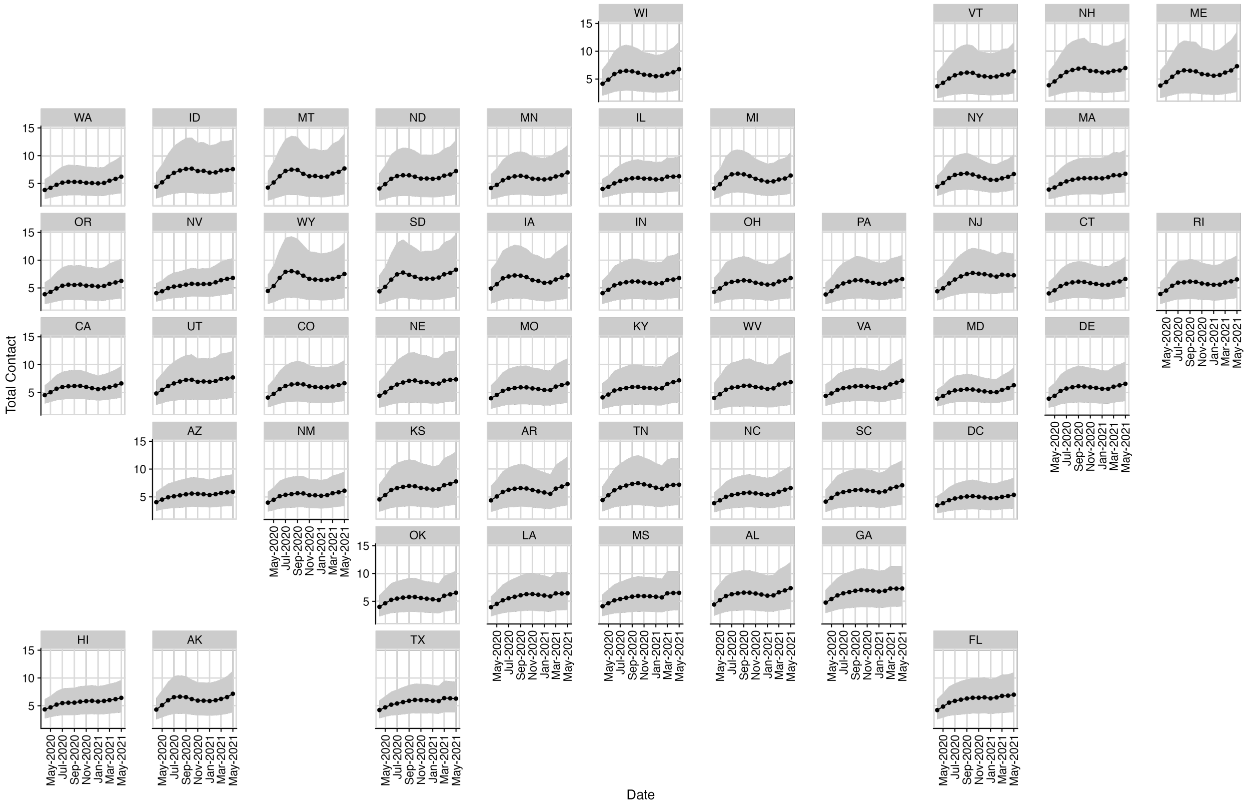

Figure B: State-specific MRP estimate of total contact (combined household and non-household contacts).

3 Additional Analyses

3.1 Internal validation check

To assess the internal validity of our method, we re-estimate our models on two subsamples. These subsamples are intended to illustrate the effects of having less data in two states: Florida and Washington. We chose these two states to illustrate partial pooling because they deviated from the overall mean in distinct ways. Florida deviates from the overall mean because there is no noticeable decline in contacts over the period between November 2020 and March 2021. Washington broadly shares similar trends over time to the overall mean, but has a lower contact rate. For the first subsample, we randomly drop all but 20% of the responses for each wave from Florida and Washington; we do not drop any data from other states. For the second subsample, we randomly drop all but 60% of the responses for each wave from Florida and Washington; again, we do not drop any data from other states. Figure C plots the the estimated contact intensity from models fit to these two subsamples and to the full sample. The figure demonstrates how partial pooling works: when there is less data available (60% subsample), the estimates are pulled towards the overall mean. When more data is available (20% subsample), the estimates more closely resemble the raw survey data. This is clearly illustrated in Florida and Washington, as estimates from 60% subsample and full sample are pulled away from the overall mean. Additionally, this provides some internal validation check for this method, as the estimates are fairly reasonable even when there is little data.

3.2 Variation in Contact Intensity

Figure D shows the standard deviation of the relative contact intensity index across states by month. This measure of how variable contact intensity was across states reveals several interesting patterns. The state-level similarity in contact intensity changed dramatically over the course of the COVID-19 pandemic. Contact intensity was most similar across states during April 2020, when social contact was lowest. When contact intensity spiked during July and August of 2020, state-to-state variation in contact intensity was highest.

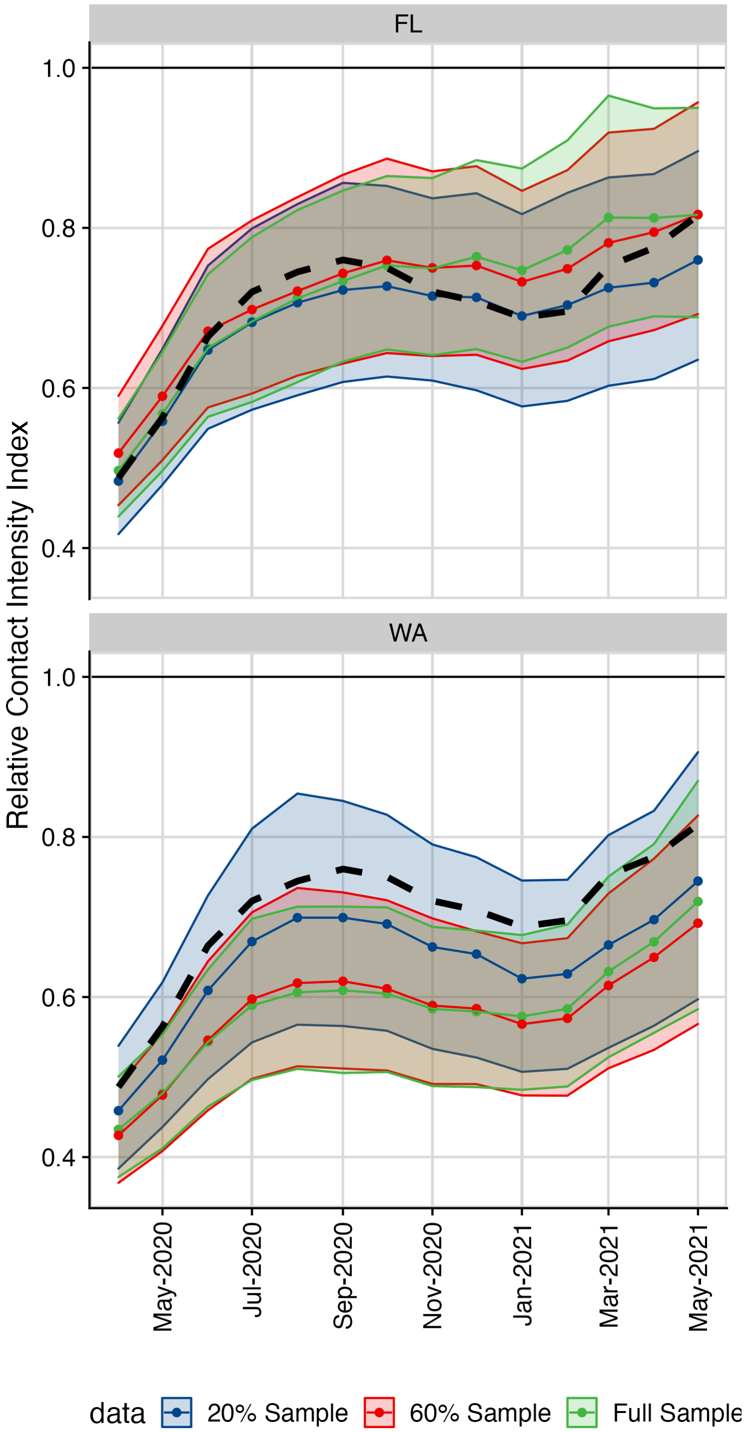

Figure C: Illustration of partial pooling. The black dashed line shows the average estimated relative contact intensity across all states.

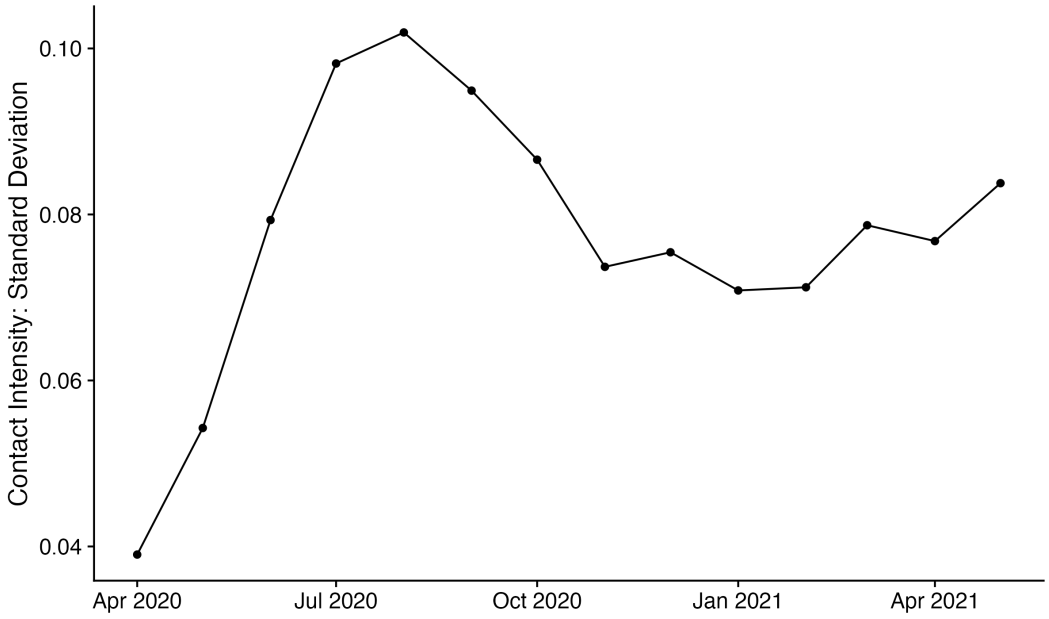

Figure D: Monthly standard deviation of contact intensity across states.

3.3 Mobility data illustration for CA

Figure E shows model input and predictions for California to give intuition about the relationship between model input and ultimate predictions of contact intensity. Specifically, panel (a) shows the first principal component of the Google mobility data and panel (b) shows the rolling three-week average of first principal component of mobility data. We used the rolling-three week average of the first principal component to smooth the data. A higher value of the first principal component indicates higher levels of mobility. Panel (c) shows the average monthly reported number of contacts in California. Panel (d) shows the implied R_0 value and panel (e) shows daily predictions of the relative contact intensity index. The cubic trend – observed in both BICS unadjusted survey data and mobility data – is also reflected in both the predicted relative contact intensity index and the monthly estimate of implied R_0.

3.4 Model without mobility predictors

While we find mobility data is only marginally predictive (see Table A), we include it in our model to highlight the model's flexible ability to incorporate state-level contextual predictors.

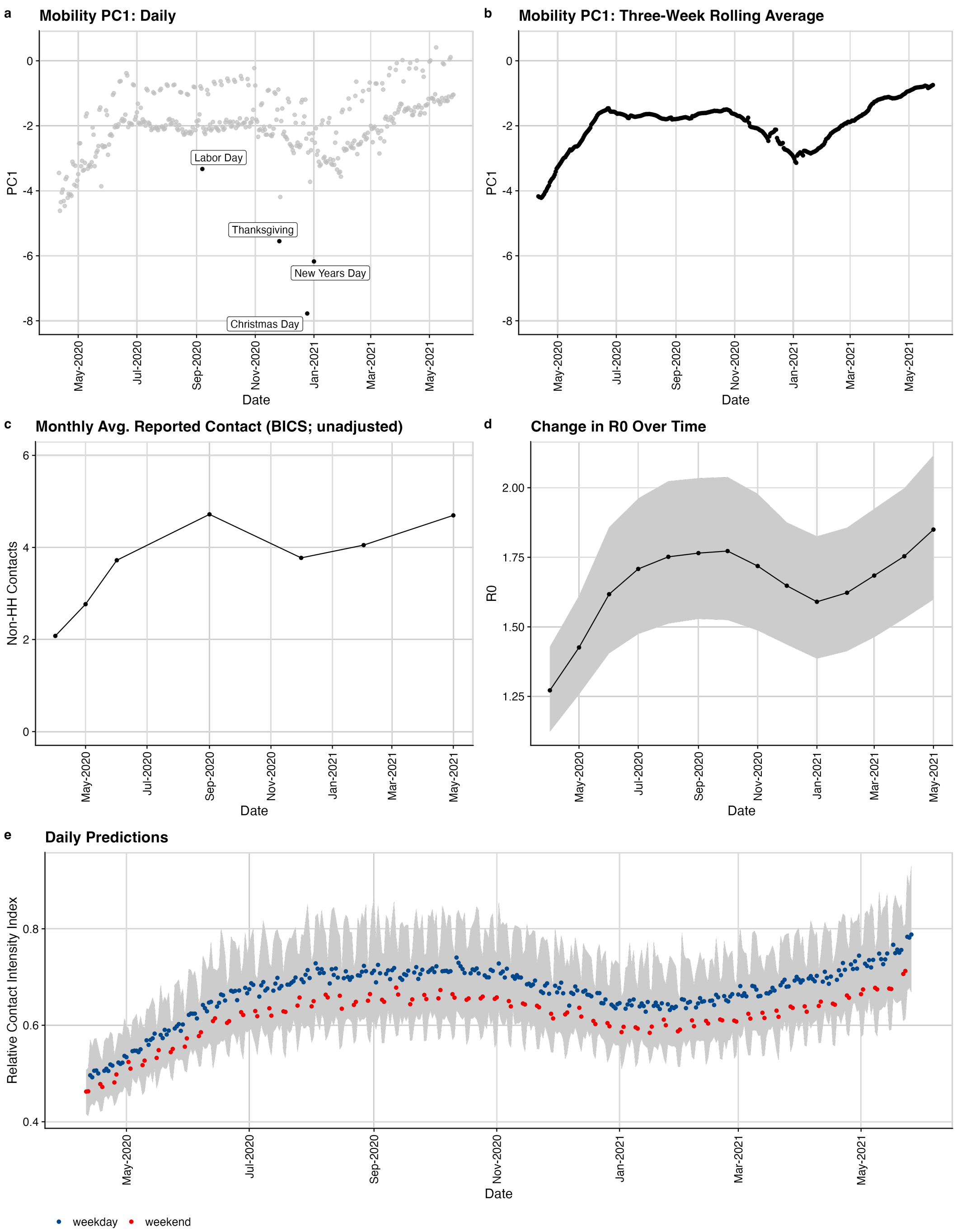

Figure E: Model input and output for state of California. Panel a shows the first principle component of seven different Google mobility signals. Panel b shows the three-week rolling average of the first principle component of seven different Google mobility signals. Panel c shows the unadjusted estimates of contact from BICS survey. Panel d shows the change in R_0 over time. Panel e shows the daily MRP model predictions.

In Figure F, we show the estimated state-level relative contact intensity index over time from a model fit *without* the mobility predictor. The estimates from our model without the

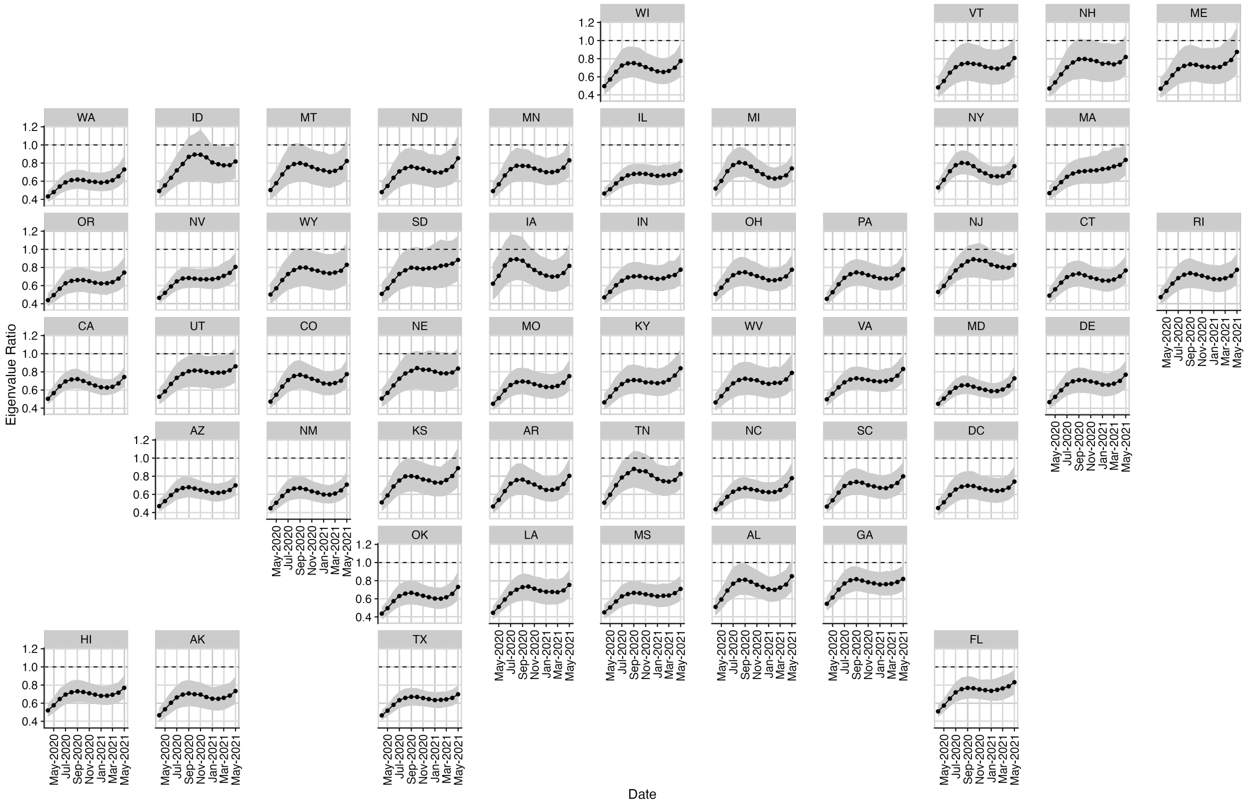

Figure F: State-level change in relative contact intensity index over time from a model without mobility data predictors.

mobility predictor closely track our estimates from the model with the mobility predictor, demonstrating that mobility data is not a prerequisite for this approach.

3.5 Model Code

All models were fit using the `brms` package in R. Full `brms` syntax below:

```
## non-household model
nonhh_model <- brm(
  formula = mvbind(alter_age_0_18, alter_age_18_25, alter_age_25_35,
  alter_age_35_45, alter_age_45_55, alter_age_55_65, alter_age_65_100) ~
    agecat +
    gender +
    hhsize +
    race +
    weekday +
    agecat:gender +
    rolling_pca_3wks + ## mobility data
    poly(day_std, 3) + ## national polynomial trend
    (poly(day_std, 3) | estimated_state), ## state level hierarchical polynomial trend
  family = 'negbinomial',
  inits = 0,
  data = df_formodel_topcode,
  chains = 4,
  cores = 16,
  backend = "cmdstanr",
  threads = threading(4),
  #  control = list(adapt_delta = 0.9),
  iter = 4000)

## household model
hh_model <- brm(
  formula = mvbind(alter_age_0_18_hh, alter_age_18_25_hh, alter_age_25_35_hh,
  alter_age_35_45_hh, alter_age_45_55_hh, alter_age_55_65_hh, alter_age_65_100_hh) ~
    agecat +
    gender +
    hhsize +
    race +
    agecat:gender +
    (1 | state),
  family = 'negbinomial',
  inits = 0,
  data = df_formodel_topcode_household,
  chains = 4,
  cores = 16,
  iter = 4000)
```

3.6 Contact Matrices by month

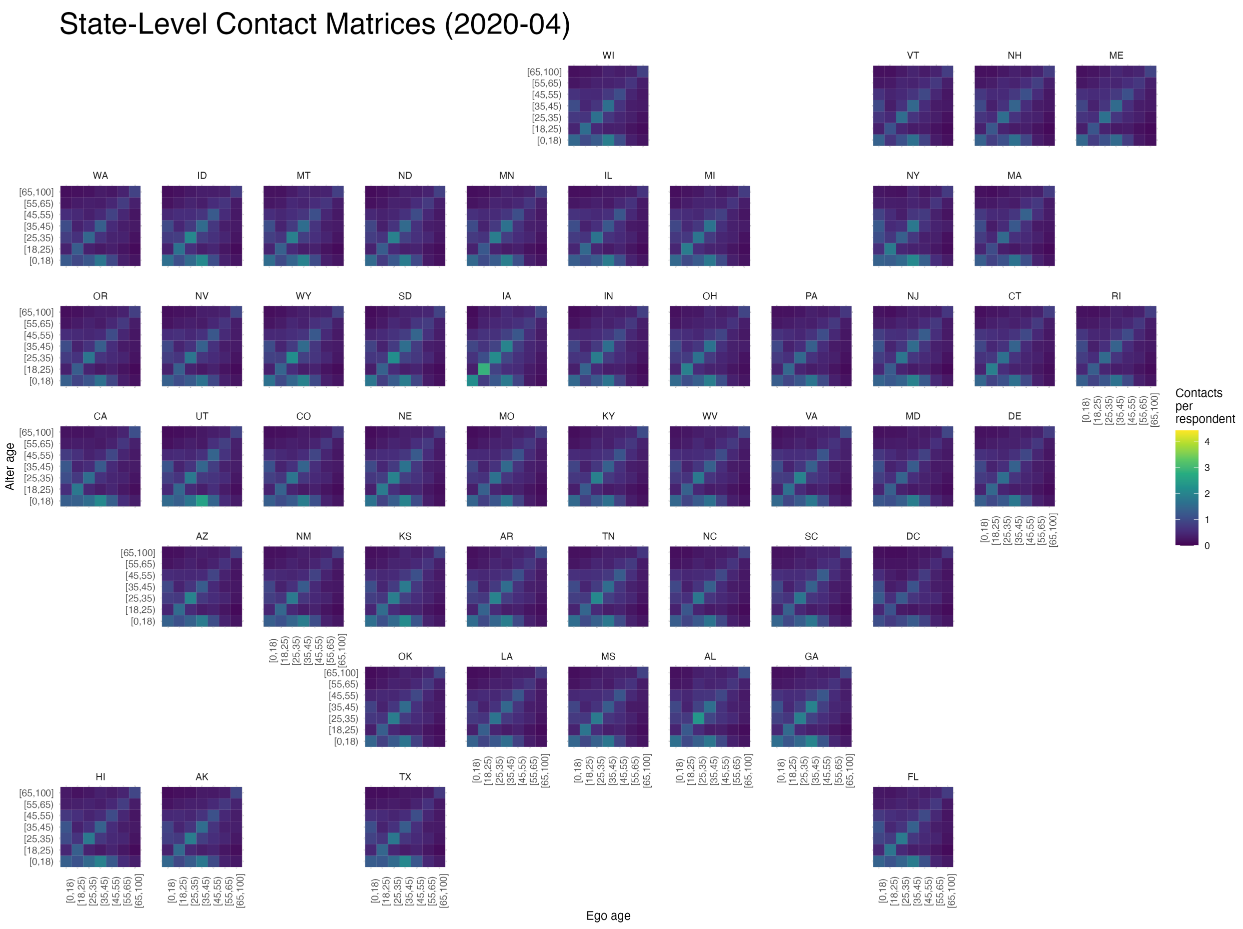

Figure G: Estimated contact matrix for 2020-04.

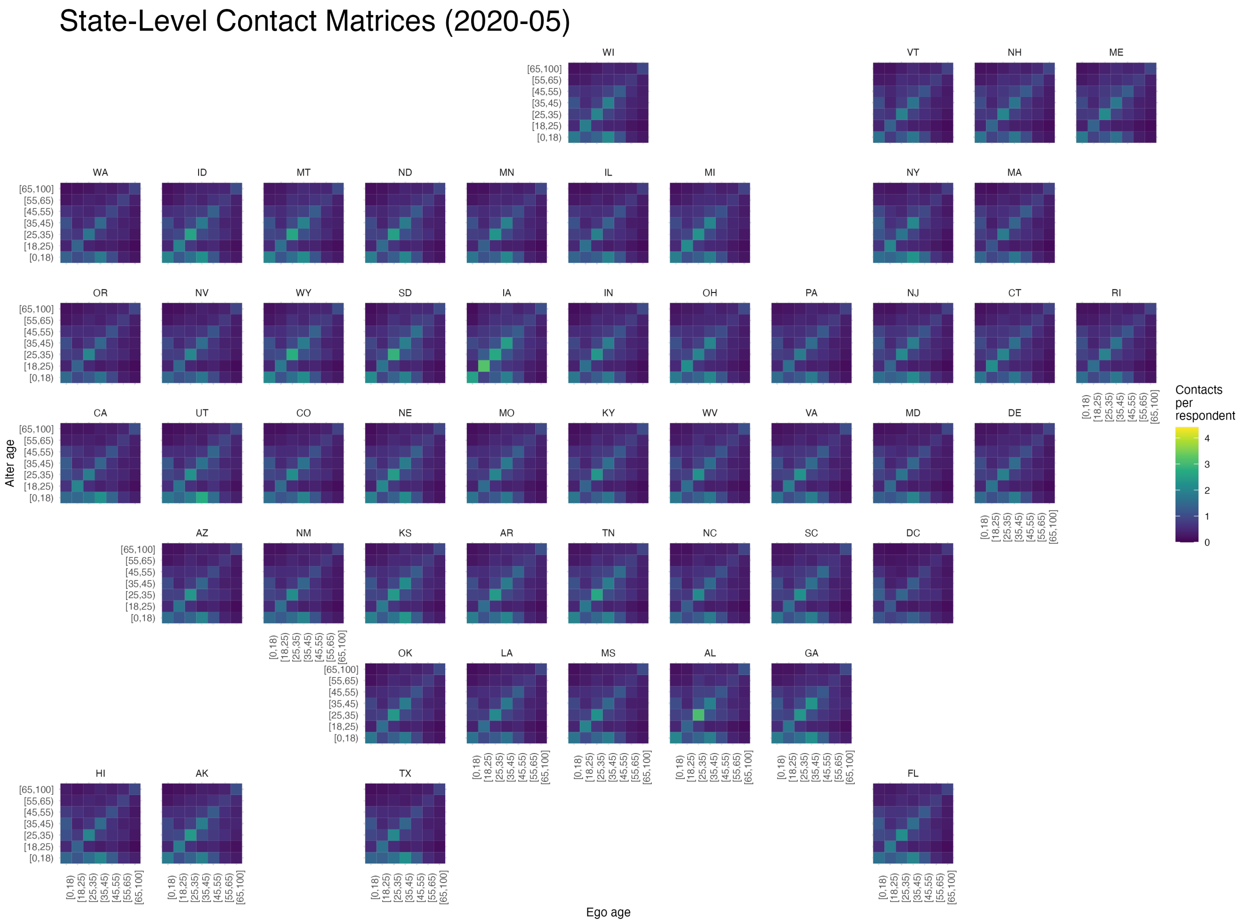

Figure H: Estimated contact matrix for 2020-05.

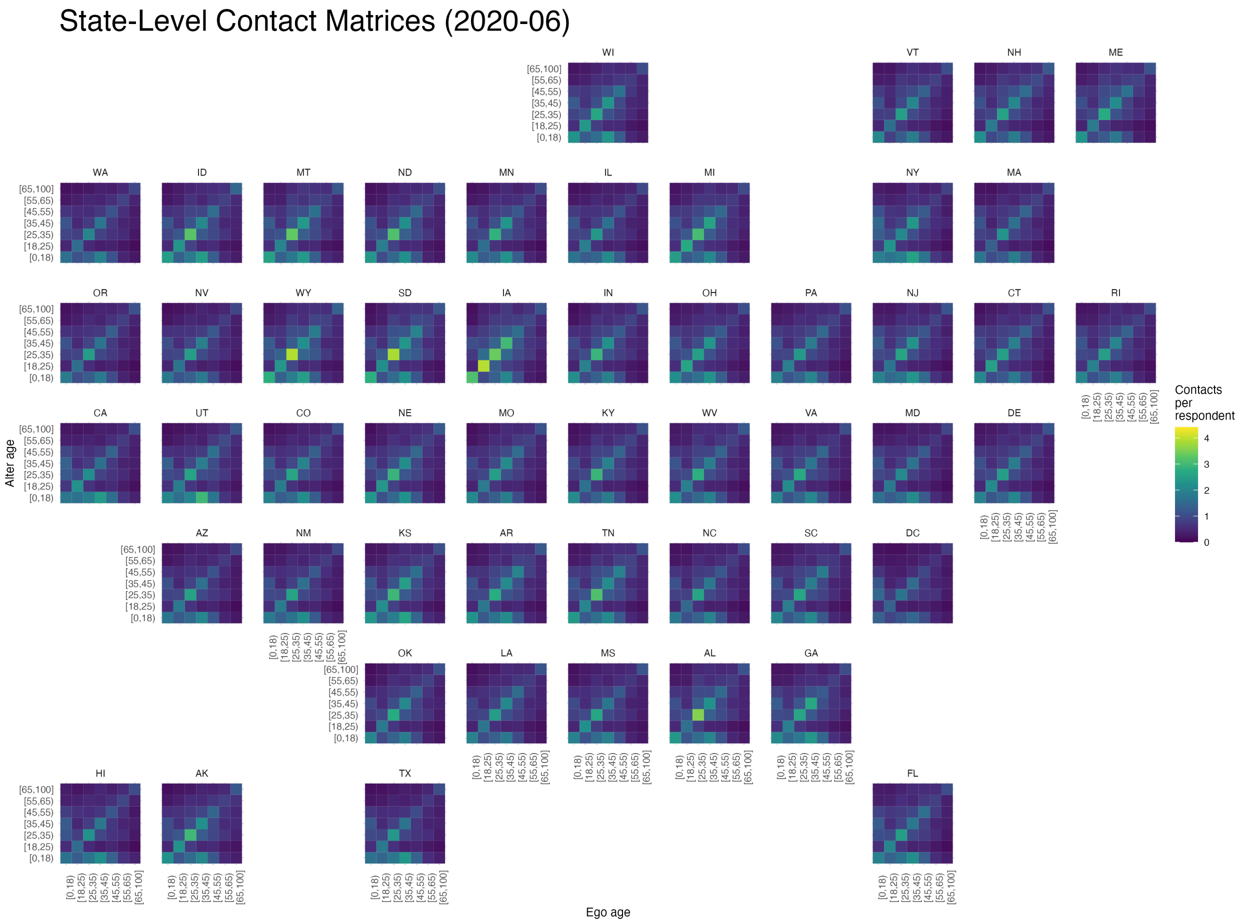

Figure I: Estimated contact matrix for 2020-06.

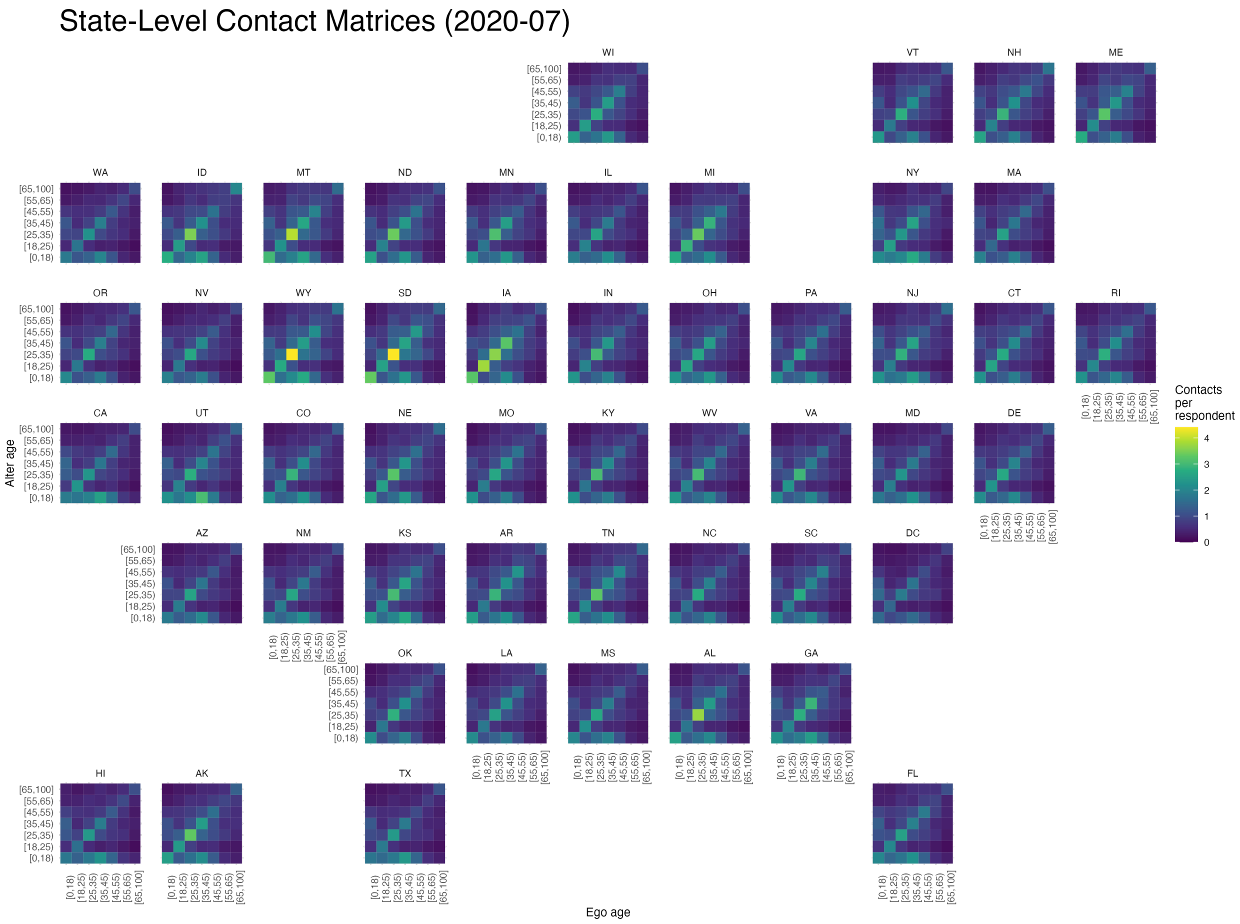

Figure J: Estimated contact matrix for 2020-07.

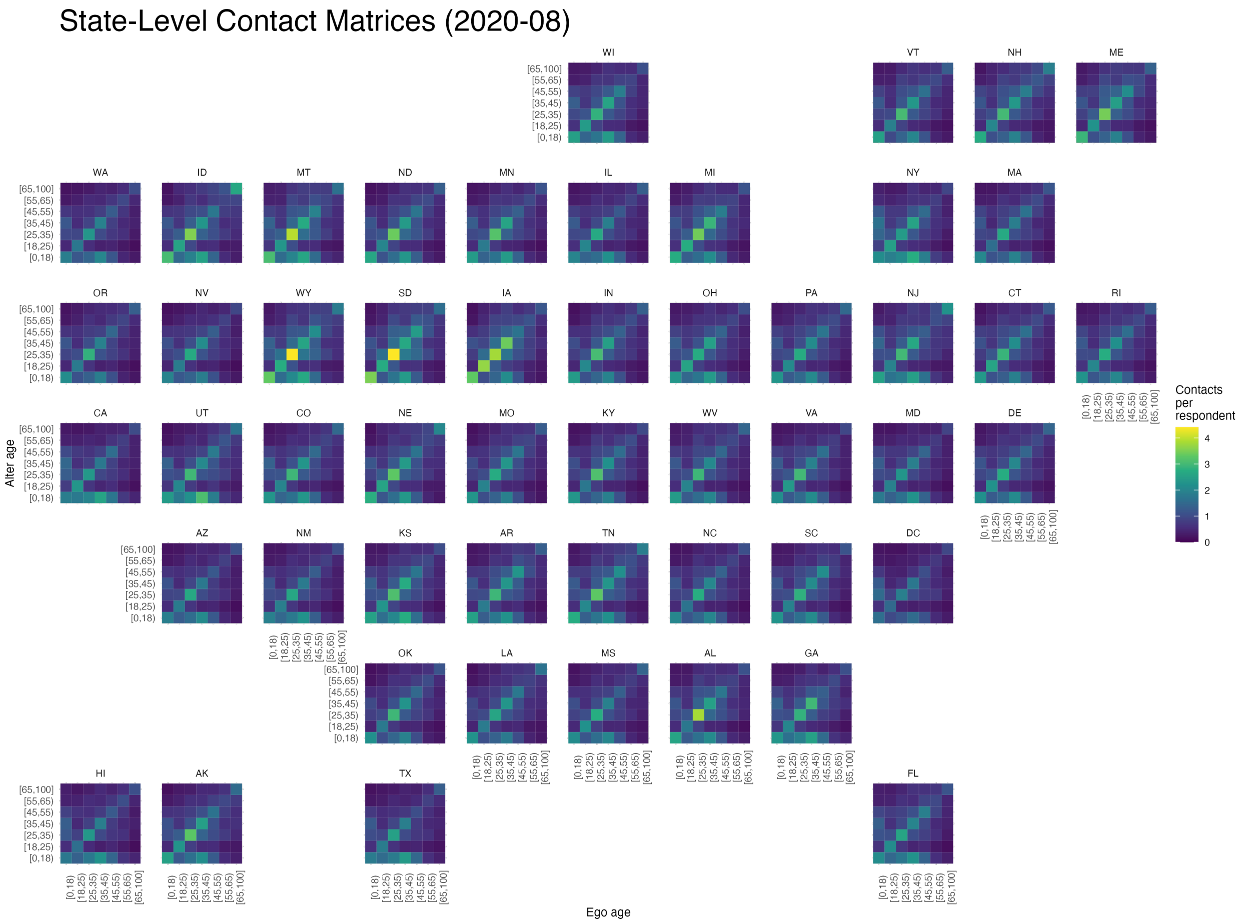

Figure K: Estimated contact matrix for 2020-08.

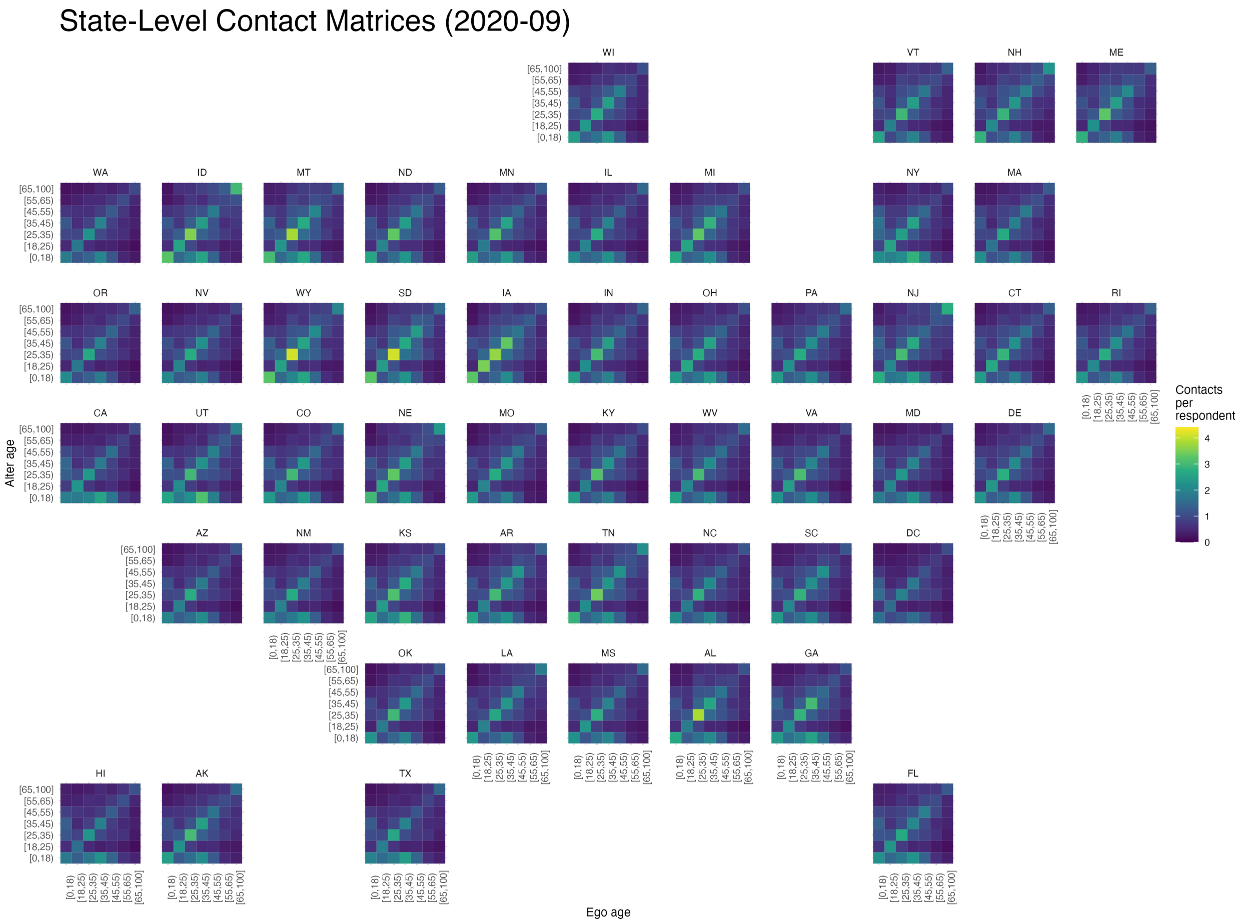

Figure L: Estimated contact matrix for 2020-09.

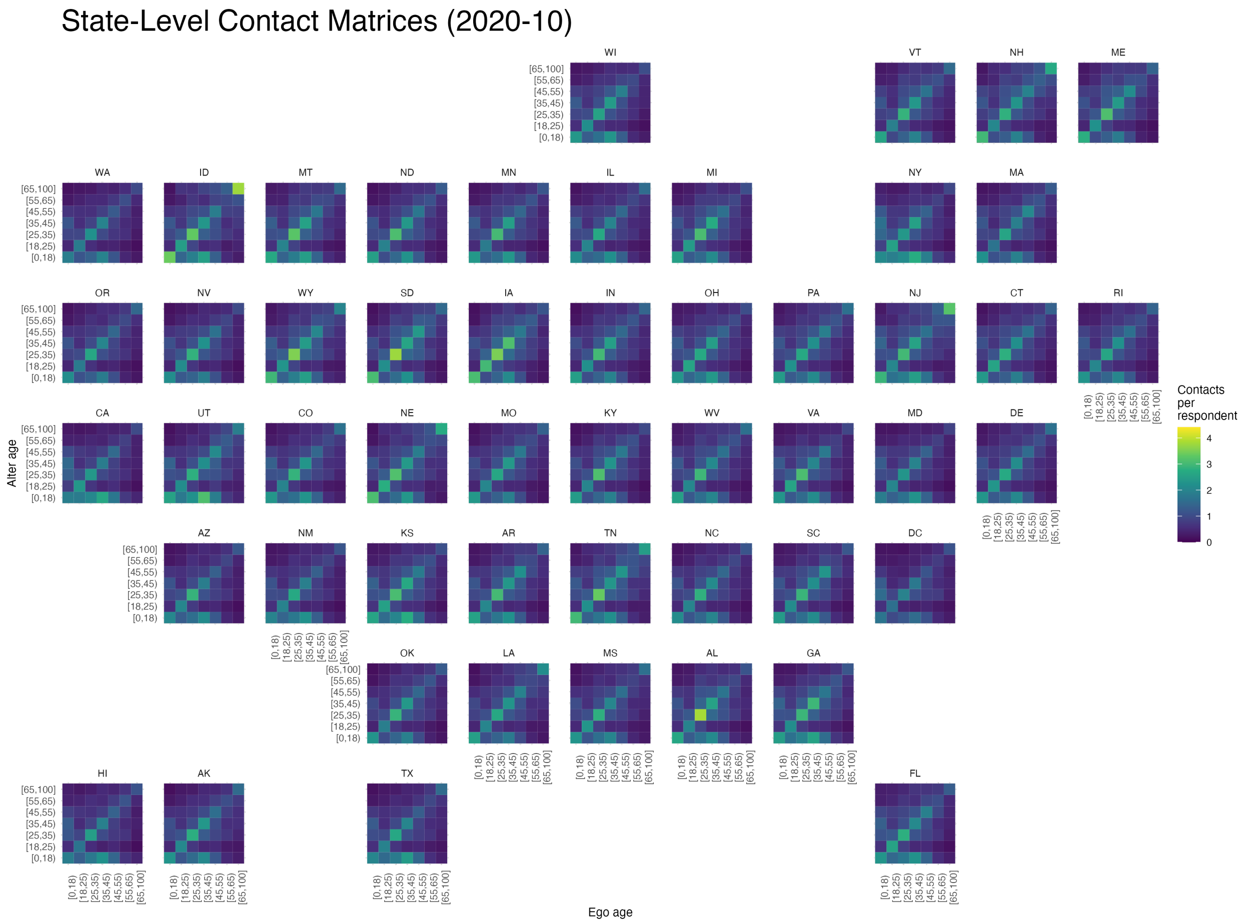

Figure M: Estimated contact matrix for 2020-10.

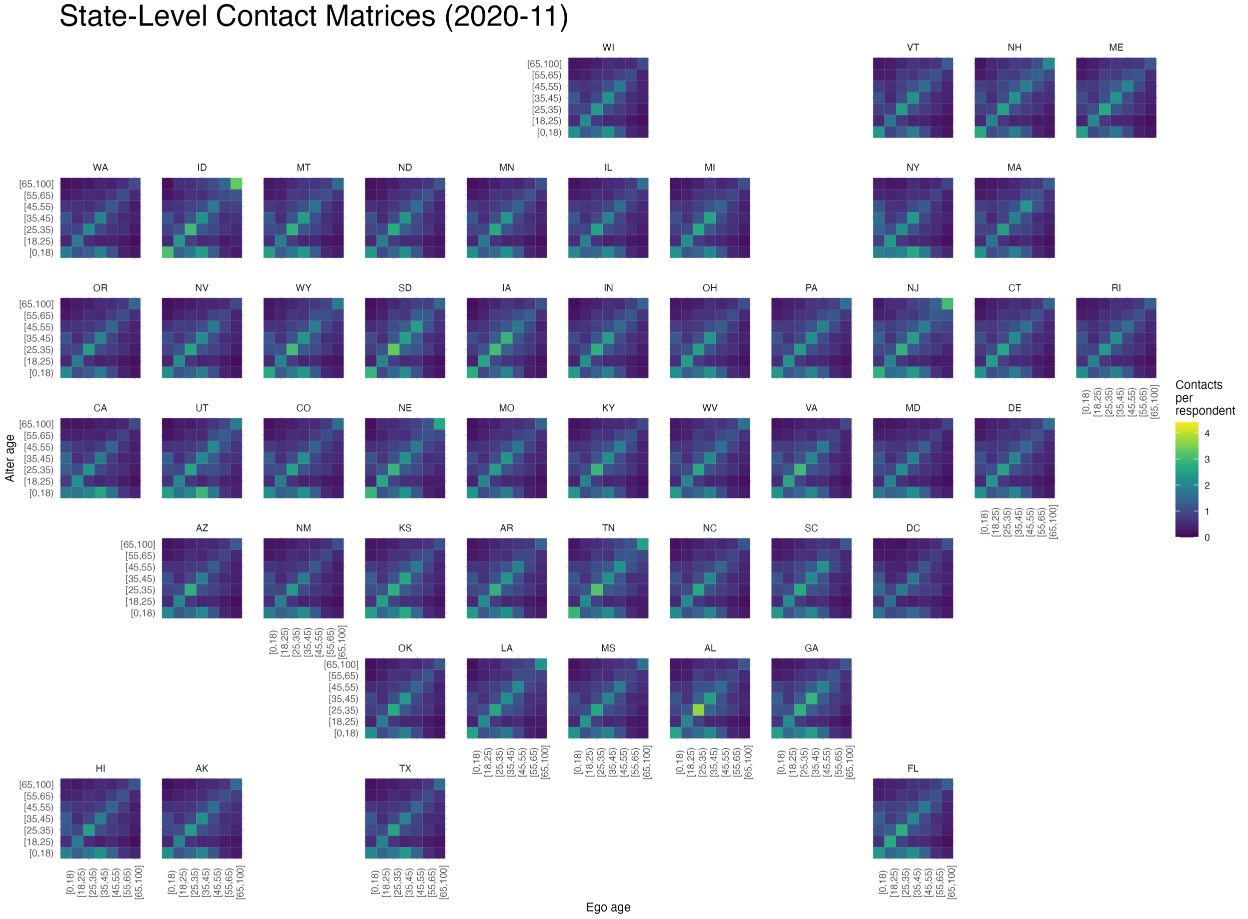

Figure N: Estimated contact matrix for 2020-11.

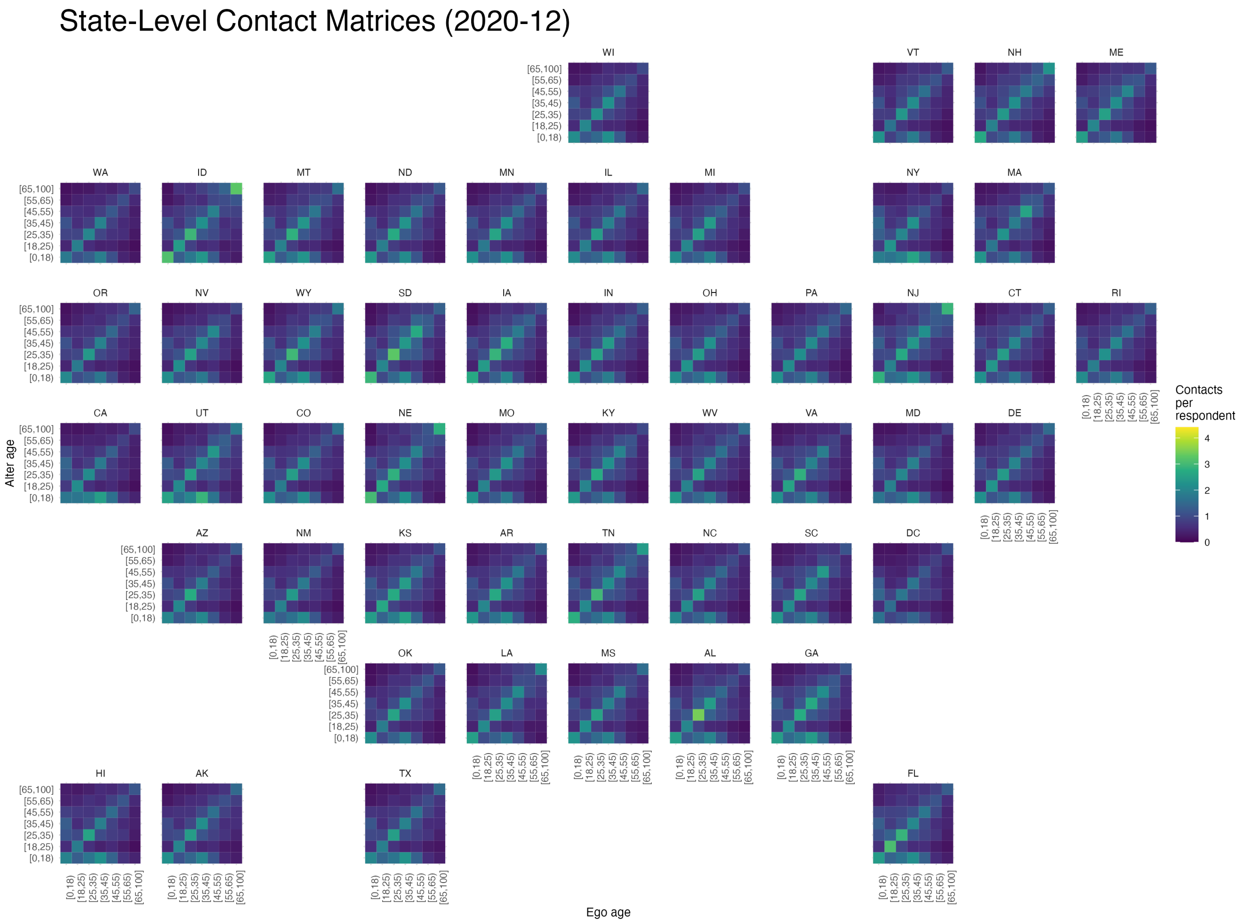

Figure O: Estimated contact matrix for 2020-12.

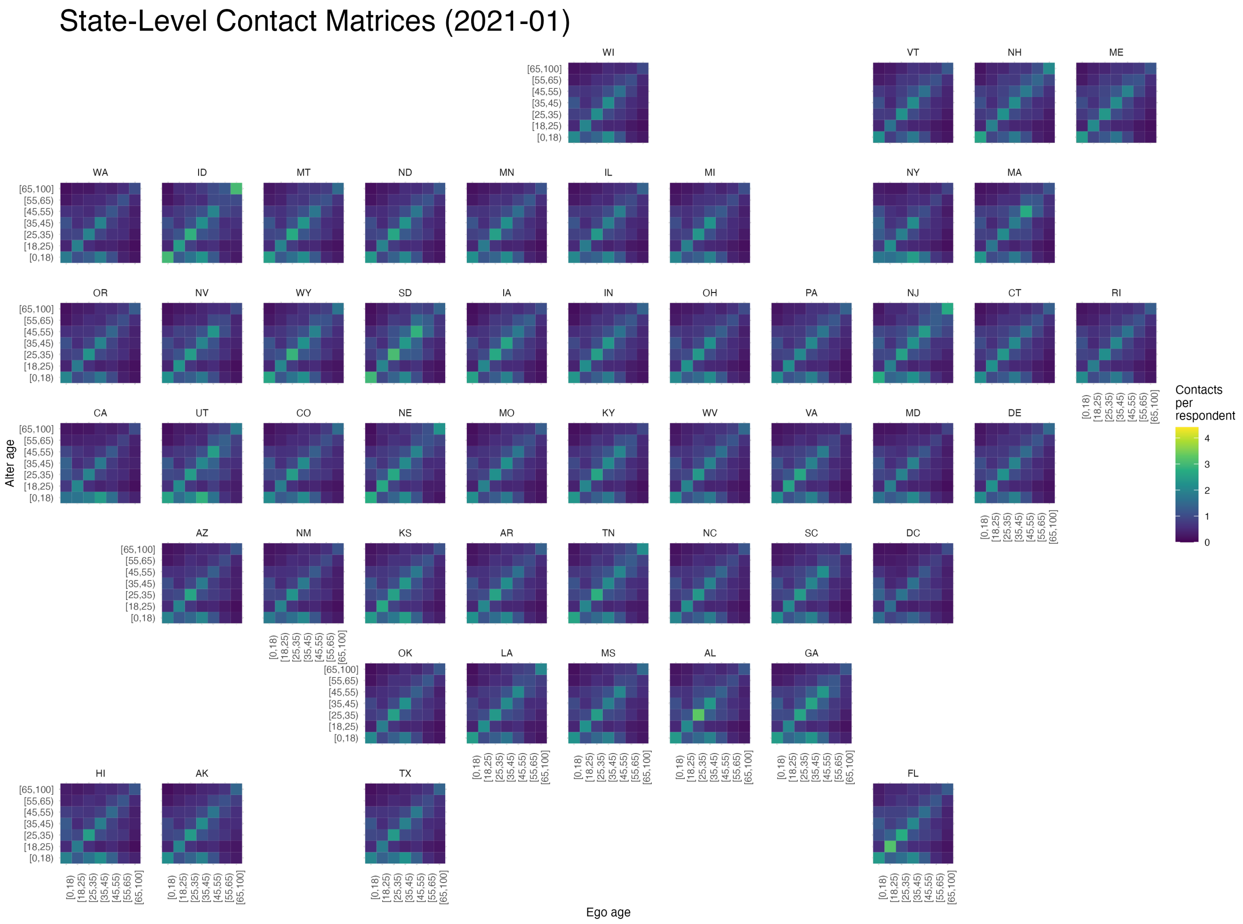

Figure P: Estimated contact matrix for 2021-01.

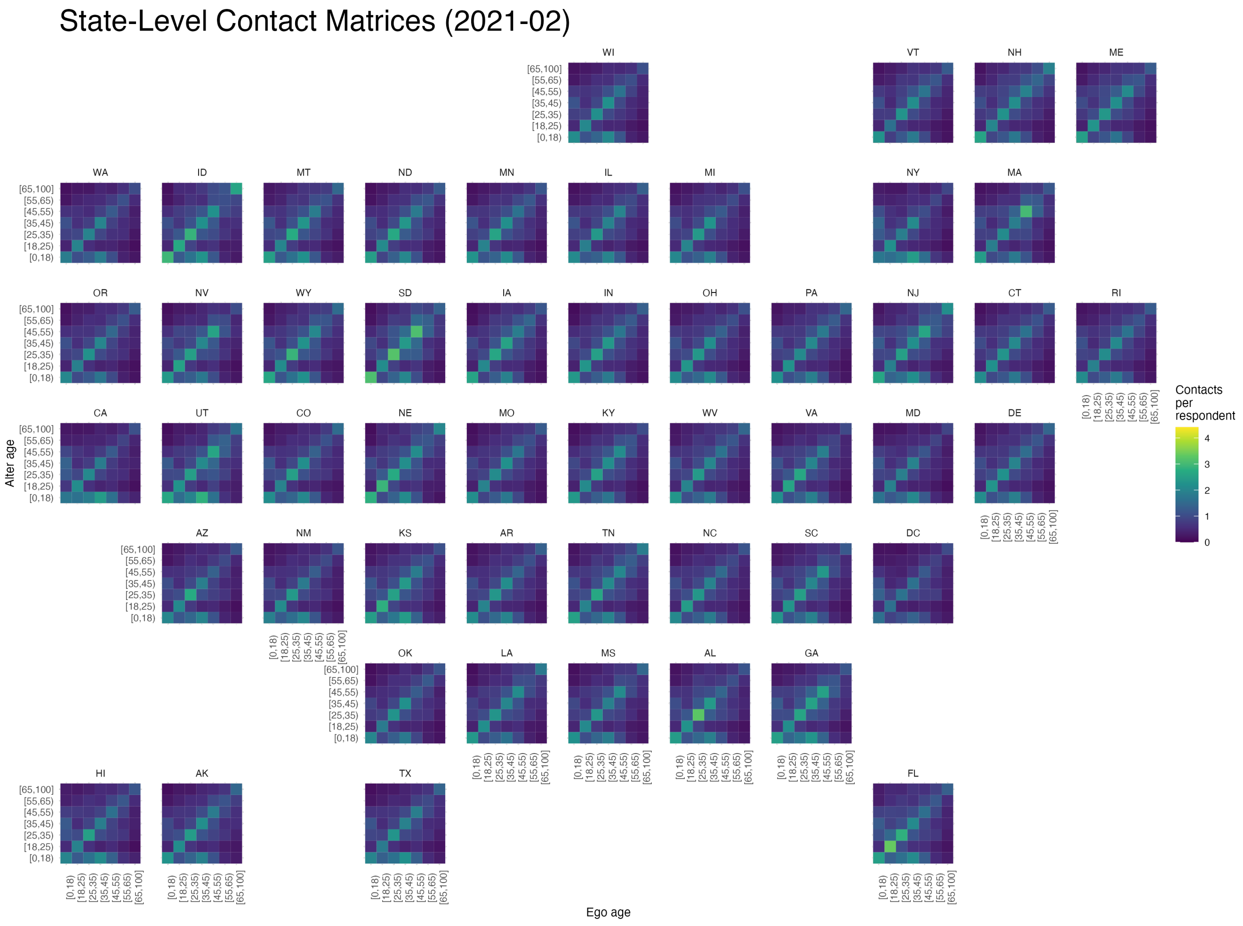

Figure Q: Estimated contact matrix for 2021-02.

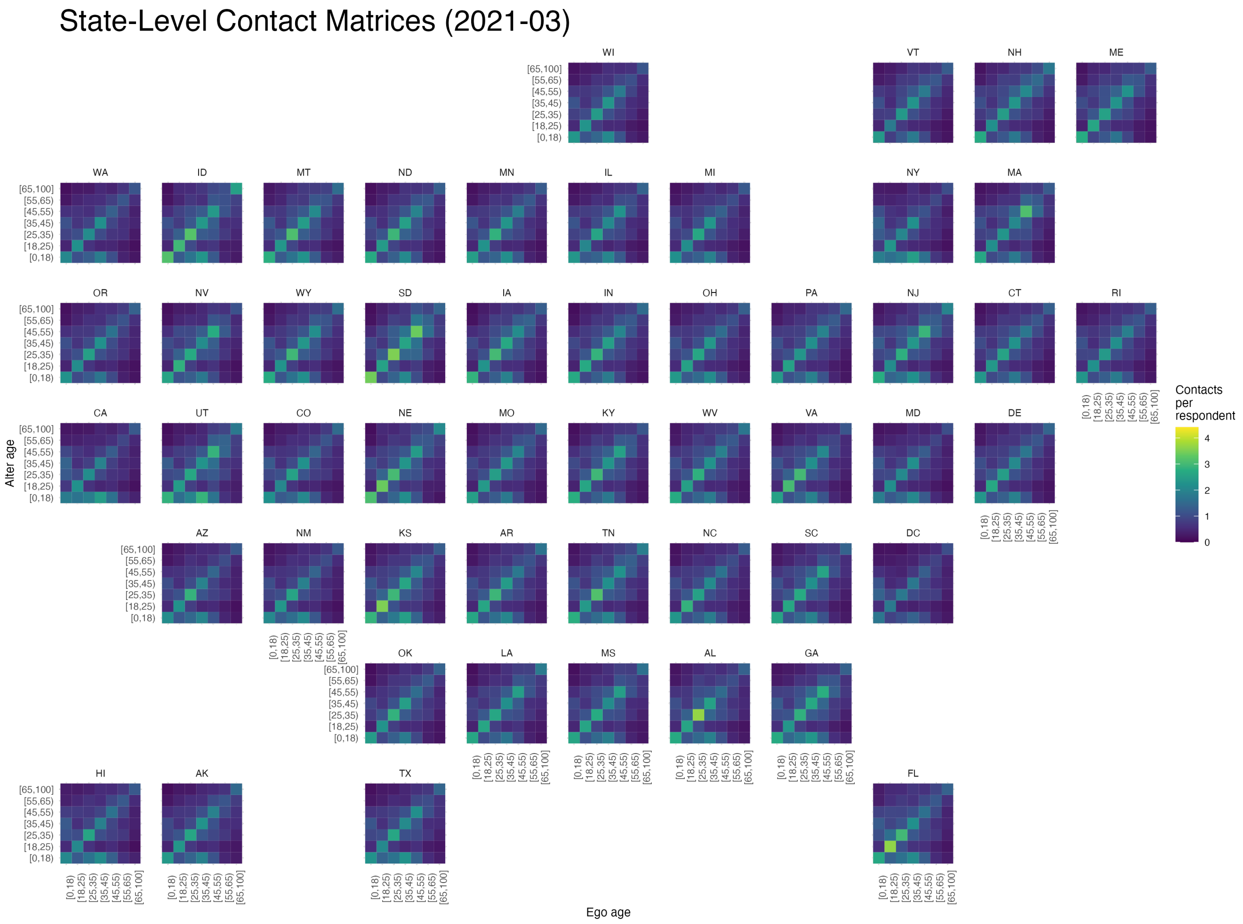

Figure R: Estimated contact matrix for 2021-03.

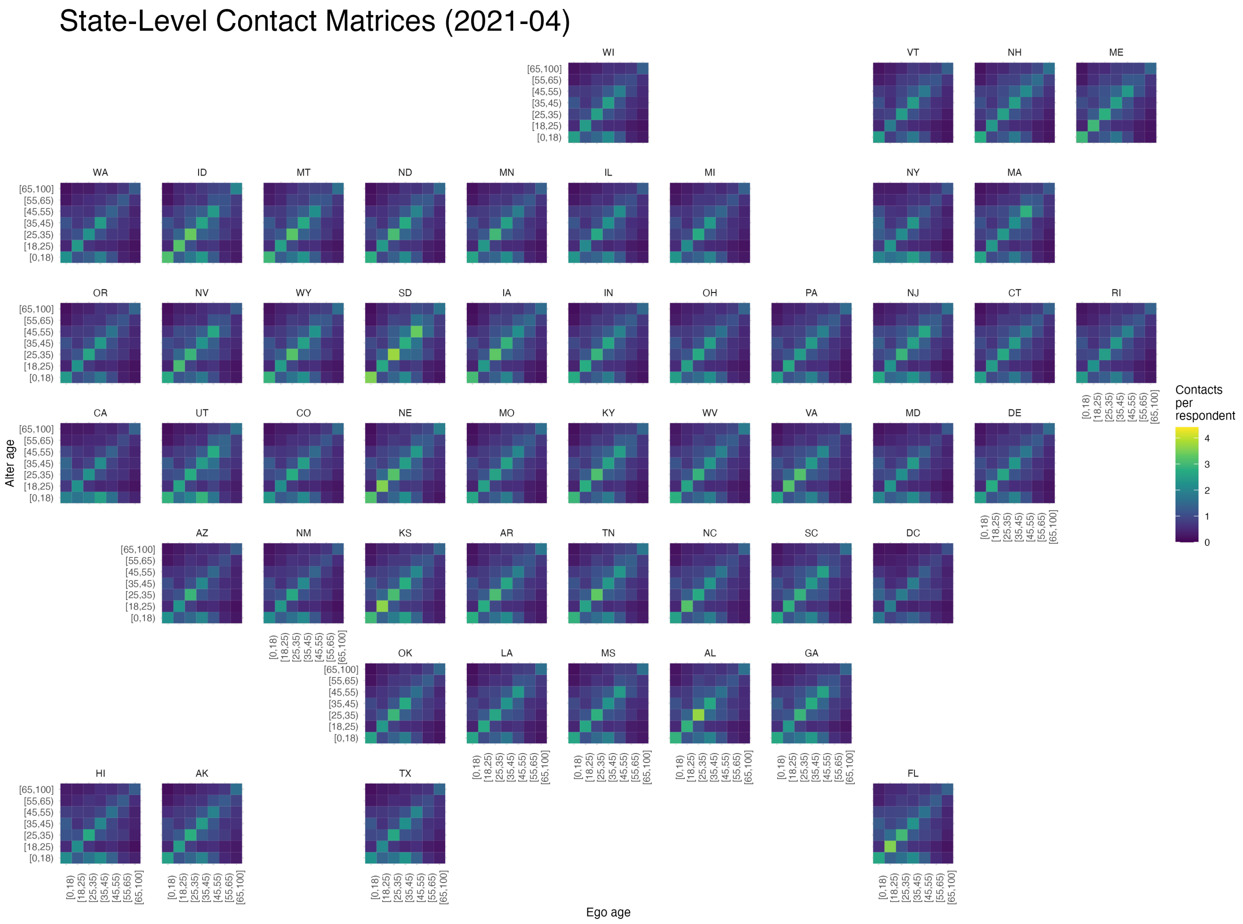

Figure S: Estimated contact matrix for 2021-04.

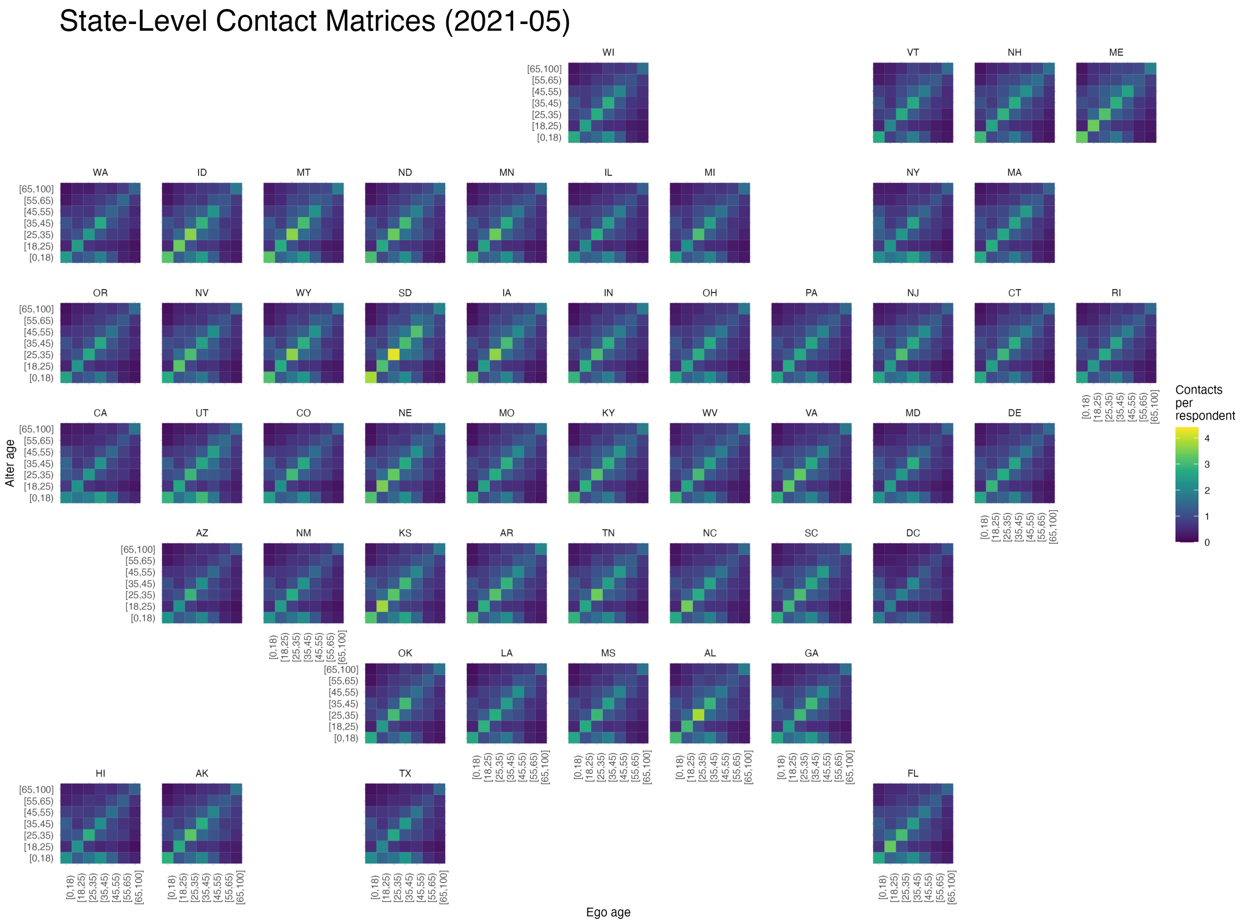

Figure T: Estimated contact matrix for 2021-05.

References

1. Bürkner, Paul-Christian. 2017. "Brms: An R Package for Bayesian Multilevel Models Using Stan." *Journal of Statistical Software, 80(1), 1-28. doi:10.18637/jss.v080.i01* .

2. Chin, Taylor, Dennis M. Feehan, Caroline O. Buckee and Ayesha S. Mahmud. 2021. Contact Surveys Reveal Heterogeneities in Age-Group Contributions to SARS-CoV-2 Dynamics in the United States. Preprint Epidemiology.

3. Davies, Nicholas G., Petra Klepac, Yang Liu, Kiesha Prem, Mark Jit, CMMID COVID-19 working group, Carl A. B. Pearson, Billy J. Quilty, Adam J. Kucharski, Hamish Gibbs, Samuel Clifford, Amy Gimma, Kevin van Zandvoort, James D. Munday, Charlie Diamond, W. John Edmunds, Rein M. G. J. Houben, Joel Hellewell, Timothy W. Russell, Sam Abbott, Sebastian Funk, Nikos I. Bosse, Yueqian Fiona Sun, Stefan Flasche, Alicia Rosello, Christopher I. Jarvis and Rosalind M. Eggo. 2020. "Age-Dependent Effects in the Transmission and Control of COVID-19 Epidemics." *Nature Medicine* 26(8):1205–1211.

4. Diekmann, O., H. Heesterbeek and T. Britton. 2013. *Mathematical Tools for Understanding Infectious Disease Dynamics*. EBSCO Ebook Academic Collection Princeton University Press.

5. Diekmann, O., J.A.P. Heesterbeek and J.A.J. Metz. 1990. "On the Definition and the Computation of the Basic Reproduction Ratio R 0 in Models for Infectious Diseases in Heterogeneous Populations." *Journal of Mathematical Biology* 28(4).

6. Fay, Robert E. and Roger A. Herriot. 1979. "Estimates of Income for Small Places: An Application of James-Stein Procedures to Census Data." *Journal of the American Statistical Association* 74(366a):269–277.

7. Feehan, Dennis M. and Ayesha S. Mahmud. 2021. "Quantifying Population Contact Patterns in the United States during the COVID-19 Pandemic." *Nature Communications* 12(1):893.

8. Feehan, Dennis M. and Curtiss Cobb. 2019. "Using an Online Sample to Estimate the Size of an Offline Population." *Demography* 56(6):2377–2392.

9. Hale, Thomas, Noam Angrist, Rafael Goldszmidt, Beatriz Kira, Anna Petherick, Toby Phillips, Samuel Webster, Emily Cameron-Blake, Laura Hallas, Saptarshi Majumdar and Helen Tatlow. 2021. "A Global Panel Database of Pandemic Policies (Oxford COVID-19 Government Response Tracker)." *Nature Human Behaviour* 5(4):529–538.

10. Hens, N., Z. Shkedy, M. Aerts, C. Faes, P. Van Damme and P. Beutels. 2012. *Modeling Infectious Disease Parameters Based on Serological and Social Contact Data: A Modern Statistical Perspective*. Statistics for Biology and Health Springer New York.

11. Holmdahl, Inga, Rebecca Kahn, James Hay, Caroline O. Buckee and Michael Mina. 2020. "Frequent Testing and Immunity-Based Staffing Will Help Mitigate Outbreaks in Nursing Home Settings.".

12. McEvoy, David, Conor G. McAloon, Áine B. Collins, Kevin Hunt, Francis Butler, Andrew W. Byrne, Miriam Casey, Ann Barber, John Griffin, Elizabeth Ann Lane, Patrick Wall and Simon J. More. 2020. "The Relative Infectiousness of Asymptomatic SARS-CoV-2 Infected Persons Compared with Symptomatic Individuals: A Rapid Scoping Review.".

13. Mossong, Joël, Niel Hens, Mark Jit, Philippe Beutels, Kari Auranen, Rafael Mikolajczyk, Marco Massari, Stefania Salmaso, Gianpaolo Scalia Tomba, Jacco Wallinga, Janneke Heijne, Malgorzata Sadkowska-Todys, Magdalena Rosinska and W. John Edmunds. 2008. "Social Contacts and Mixing Patterns Relevant to the Spread of Infectious Diseases." *PLoS Medicine* 5(3):e74.

14. Vehtari, Aki, Andrew Gelman and Jonah Gabry. 2017. "Practical Bayesian Model Evaluation Using Leave-One-out Cross-Validation and WAIC." *Statistics and Computing* 27(5):1413–1432.

15. Viceconte, Giulio and Nicola Petrosillo. 2020. "COVID-19 R0: Magic Number or Conundrum?" *Infectious Disease Reports* 12(1):8516.

16. Wallinga, Jacco, Peter Teunis and Mirjam Kretzschmar. 2006. "Using Data on Social Contacts to Estimate Age-specific Transmission Parameters for Respiratory-spread Infectious Agents." *American Journal of Epidemiology* 164(10):936–944.
