# Supplementary material for: Novel estimates reveal subnational heterogeneities in disease-relevant contact patterns in the United States
Source: PLoS Comput Biol. 2022 Dec 2;18(12):e1010742. doi: 10.1371/journal.pcbi.1010742 (PMC9749998; doi:10.1371/journal.pcbi.1010742)
Supplement: Attachment — Submitted filename: bics_mrp_response_letter.pdf [file pcbi.1010742.s002.pdf]

Submission #PCOMPBIOL-D-22-00658 for PLOS Computation Biology
Response to Reviewers

Dear Dr. Daniel B Larremore and the Editorial Team,

Thank you for giving us the opportunity to submit a revised draft of our manuscript, “Novel Estimates Reveal Subnational Heterogeneities in Disease-Relevant Contact Patterns in the United States” for publication in PLOS Computational Biology. We thank the reviewers for their careful read of the manuscript and constructive advice for improvement. We have revised the manuscript in line with the comments of the reviewers, and we believe it is now a stronger, more focused paper. Among the most important changes:

(1) We restructured the paper so the methods section comes before the results and discussion sections. We have also expanded the methods section to more clearly express the specific methodological contribution of this paper.
(2) To validate our method, we reran our MRP model on random subsamples including only 20%, 60%, and 100% of the original data for certain states. This analysis illustrates the impact of pooling and provides an internal validation of our method.
(3) We fit a new model incorporating the Oxford Stringency Index (OSI), which captures the strictness of “lock-down” policies at the state-level that primarily restrict people's behavior. While we found that OSI did not improve model performance in our setting, we now include Supporting Information Section C.2 discussing the potential of adding non-pharmaceutical intervention predictors in other applications.
(4) We recalculated the R0 values assuming age-specific transmission patterns.

Below, we have additionally provided a point-by-point response (in blue) to each suggestion made by the reviewers.

Editor: *All three reviewers found that this submission represents a substantial, interesting, and valuable contribution. They also raised a number of constructive points that could strengthen the paper, and, were those to be addressed, I would be excited to consider a revised submission. One common thread raised by R1 and R2 is validation. Here, the reviewers make substantially different suggestions; I suggest that the authors consider whether the constructive goals of both reviewers might be reasonably achieved even if only one approach to validation is followed.*

Reviewer #1:

The manuscript “Novel Estimates Reveal Subnational Heterogeneities in Disease-Relevant Contact Patterns in the United States” presents a novel methodology to estimate age-specific contact patterns at the subnational level. The method is based on multilevel regression with post stratification and can flexibly account for cell-level estimates with a very low number of counts. I think that this work has a good potential and addresses an important open question, that is the one to estimate sub-national social contact data starting from national surveys.

We thank the author for the overview and the helpful suggestions for improvement.

However, I think that there are several methodological issues that need to be addressed more carefully.

The approach of the authors uses a flexible level of pooling, with estimates that are closer to the survey response when many observations are available and estimates that are pulled away from survey response towards the mean when less data is available. This is indeed a very interesting and promising approach but I think that the authors are neglecting to consider the effect of Non Pharmaceutical Interventions (NPIs) on social contacts. Several studies [Gimma et al. 2020,Coletti et al. 2020, Liu et al. 2021] have shown that social distancing directions can reduce the number of social contacts substantially. It would seem that the hierarchical model is pooling data not taking the effect of NPIs into account as these are not included in the hierarchical model. Note that the inclusion of Google mobility is not sufficient to account for this, as Google mobility is not able to capture the full extent of contact reduction [Tomori et al. 2021]. This is even more important when looking at the contacts for the younger age class (0-18): When schools are closed, pooling information from situations in which schools are open (or vice-versa) would most likely produce wrong estimates. There are several possibilities to include the NPI In the model, for example including the Oxford stringency index as a possible covariate of the model (Oxford 2020), to avoid pooling together information that is not homogeneous with respect to NPIs.

We thank the Reviewer for this suggestion. The Reviewer is correct that our model is not taking into account the effect of NPIs. In response to this suggestion, we tried adding the Oxford Stringency Score as an additional covariate in the model and found that, in this specific setting, the model's performance did not improve. According to LOO-CV, there was a slight, non-statistically significant decline in model performance (see Table 3). However, we agree with the Reviewer's broader point that data about NPIs could be very valuable to improving similar models in many settings. To make this clear, we added a section into our Supporting Information discussing how NPIs could be added to our method in other settings (see Supporting Information, page 12).

Also, the authors mention that they develop a dynamical model that pulls information from different time points. Although this has the advantage of making use of the full information it is not clear to me whether there is any preference among pulling "over time" or pulling "over demographic strata." To be more clear, to estimate data for women aged 25-35 living in New York in April, does the model prefer information from different time points (e.g.women aged 25-35 living in New York in July) or from different locations (women aged 25-35 living in New Jersey in April)? If this is selected from the model, it should be clarified and discussed also in relation to the previous point (different NPIs taking place over time in different locations).

We thank the Reviewer for this very interesting question. Upon reflection, we have concluded that there is not a simple answer: the model is pooling both over time and over demographic strata. However, pooling over demographic strata comes directly from hierarchical terms in the model, while pooling over time comes from state-specific polynomial time trends whose coefficients are hierarchically pooled. We note that applying MRP in dynamic settings is an emerging area of research (Gelman et al., 2018), and we hope that as the field develops, deeper intuition will follow. We also note that new insights about dynamic MRP could readily be incorporated into the modeling framework we present in this paper.

Again related to the younger age class it seems to me that the authors are relying on several assumptions to estimate the contacts of children. I think that an interesting sensitivity analysis would be to exclude the 0-18 age class from the model, in order to estimate contact matrices for adults only and assess to what extent a "zero assumption analysis" differs from the main one.

We agree that because our sample does not include those age (0-18], our analysis relies on several assumptions to estimate the contacts of the (0-18] age class, most notably the reciprocity assumption. However, the assumptions we have to make for the (0-18] age class do not affect our estimates for the older age classes. This is because our model fits seven parallel negative binomial models that separately estimates the number of contacts for each age class. When we drop the (0-18] age class from our model, we drop all contacts with that age class, but this has no impact on contacts with other age groups. The resulting estimated contact matrices for adults only are virtually identical.

The authors assume no age-specific differences in susceptibility and infectivity. This has been proven to be a wrong assumption by several works [Davies et al. 2021, Franco et al. 2021] and I think that a proper sensitivity analysis with respect to this assumption would be needed. Even better, the authors may drop this assumption and include one (or more, as sensitivity analysis) set of age-specific values for susceptibility and infectivity.

We appreciate this suggestion, and agree that dropping the no age-specific differences in susceptibility and infectivity assumption makes sense. We have added a new section to the Supporting Information calculating R0 using an age-structured deterministic compartmental model with age-specific transmission patterns from Davies et al. 2021 (see Supporting Information, page 8).

Finally, I think that some validation of the method would be useful. Although already used in other settings, MRP has never been applied to social contact data. A sensitivity analysis aimed at quantifying the impact of MRP for different levels of data sparsity would help the reader to appreciate the strength of the method. This could be done, for example, generating from the original dataset a subsample that for one given state and time point includes only (e.g.) 20%, 40%, 60% and 80% of the original data. Applying the MRP to these subsamples may help the reader to have an idea of the impact of pooling. Also, having that the pooled results for low percentage sub-sample (i.e. including just a fraction of the original data) are in line with the full data would provide a good sanity check of the method.

We appreciate this concern and thank the Reviewer for the suggestion. We have implemented the Reviewer's suggestion, and show the results in Supporting Information Section 3.1. This exercise provides some (internal) validation of our method, both illustrating the idea of partial pooling and providing a good sanity check for our method. In addition, we have expanded our discussion of the importance of validating our method in the future, specifically by comparing our estimates against reliable contact surveys that collected state-level contact patterns for one or more states (see page 21).

Page 13, line 214: "the majority of countries lack nationally representative contact surveys". Although this is true, a large number of new surveys have been developed in recent years in developed [Hoang et al.2019] as well as developing [Mousa et al. 2021]. Also data for covid-19 have been extensively collected [Liu et al. 2019]. The reader should be made aware of this large number of social contact studies available.

We are grateful to the Reviewer for pointing us to these additional contact studies. We now cite these studies (see page 6).

Page 14 Line 262: "while household contacts remain relatively stable." In general, household contacts tend to go down when social distancing rules are relaxed. Could the authors take into account this trend as well?

Thanks for the suggestion. While we are able to observe how household size changes over the course of the pandemic in our data (no downward trend), we are not actually able to observe whether contacts within the household go down as social distancing rules are relaxed. We assume all household members are household contacts. We have added a sentence about future work dynamically modeling the household contacts (see page 21).

Page 15 Line 284 "weighted": do the authors here mean the weights of the post-stratification?

We appreciate the question and agree the phrase "weighted" is confusing. We now clarify that we are referring to the total number of contacts in each age group, which were calculated by up-weighting the detailed contacts (see page 9). We thank the Reviewer for pointing this out.

In conclusion, I think that the authors should address these issues, adapt the methodology accordingly, and re-submit their work.

We thank the Reviewer for their helpful comments and constructive suggestions for improvement.

Bibliography

Coletti et al: "CoMix: comparing mixing patterns in the Belgian population during and after lockdown ". Scientific reports 2020.

Davies et al: "Age-dependent effects in the transmission and control of COVID-19 epidemics". Nature Medicine 2020.

Franco et al:"Inferring age-specific differences in susceptibility to and infectiousness upon SARS-CoV-2 infection based on Belgian social contact data". PLOS Computational Biology 2022.

Hoang et al:"A Systematic Review of Social Contact Surveys to Inform Transmission Models of Close-contact Infections". Epidemiology 2019.

Gimma et al: "Changes in social contacts in England during the COVID-19 pandemic between March 2020 and March 2021 as measured by the CoMix survey: A repeated cross-sectional study". PLOS Medicine 2022.

Hoang : Liu et al: "Rapid Review of Social Contact Patterns During the COVID-19 Pandemic ". Epidemiology 2021.

Mousa et al: "Social contact patterns and implications for infectious disease transmission – a systematic review and meta-analysis of contact surveys ". ELife 2021.

Tomori et al: “Individual social contact data and population mobility data as early markers of SARS-CoV-2 transmission dynamics during the first wave in Germany-an analysis based on the COVIMOD study”. BMC Medicine 2021.

Reviewer #2:

This article is both timely and informative, employing a sophisticated analysis that ties together a number of strands in MRP research in a way that is very useful for other researchers working on similar problems. I have only a few minor suggestions for improvement:

We thank the Reviewer for their careful read of the paper and detailed suggestions for improvement.

1. It would be useful to discuss ways in which the results might be validated. For example, could the household age-structure estimates be meaningfully compared with household age-structure estimates taken directly from the ACS? Even if such validation is not included in the article, it would be useful to discuss how this might be approached in future research.

We appreciate this concern, and we expanded our discussion in the discussion section about the way in which this approach could be validated. As we post-stratify our estimates to the ACS on household age structure, we unfortunately cannot use ACS household age-structure to validate our estimates. We do use the approach suggested by Reviewer 1 as a sensitivity analysis to bolster the credibility of our method. However, we recognize the best way to externally validate these estimates is to compare them to estimates from a high-quality, state-level contact study, which ideally captures several states during several time periods. We expand on this in our discussion section (see page 20).

2. I find Equation 9 in the supplemental materials (section 6.2) very confusing the way it is written and the way the variables are defined. I realized that this equation is being used in a lot of the contact matrix literature, but it is not clear to me why the i's and j's are used differently on the left-hand side (chatij is mean contacts of age group i with members of age group j) and the right hand side (where cji is used for this and where Nj is defined as the size of age-group i). Would it not be easier to interpret as:

*c_{hat}ij = 1/2Ni * (cij Ni + cji Nj)*

Where c{hat}_{ij} and cij are both defined in terms of mean contacts for group i with group j, Ni is defined as the size of group i, etc.? There may be a good reason for writing it the way you have it, but I would at least consider the advantages of writing it in this more intuitive way.

Thanks very much for suggesting this more intuitive notation. We agree that the notation the Reviewer suggests is more intuitive, and we have rewritten this equation in line with the reviewer's suggestion (see page 6 and Supporting Information, page 4 and 5).

Review #3:

Thank you for the opportunity to review this manuscript. The authors identify an important issue regarding the use of contact studies for the modelling of infectious diseases: there is often substantial geographical heterogeneity in contact behaviour which is not reflected in national-level contact surveys.

The authors present a technique (MRP) which has been successfully used in other fields and apply it to national contact studies to obtain sub-national contact estimates in the context of the COVID-19 pandemic in the USA.

The research is mostly sound and well-presented, but would benefit from a few additional improvements before it is suitable for publication. Note that I am not an expert in hierarchical Bayesian modelling, so I cannot provide extensive comments on the use of this method here.

We thank the Reviewer for their careful read of the paper and detailed suggestions for improvement.

Comments:

Abstract:

"To overcome this challenge, we introduce a flexible model that can estimate age-specific contact patterns at the subnational level by combining national-level interpersonal contact data with other locality-specific data sources": if the word count for the abstract allows it, it would be good to refer to the methods you will be using here.

We thank the author for this suggestion. We have now clarified in the abstract our approach will use multilevel regression with poststratification (MRP).

Introduction:

Line 16: I don't think the heading 'author summary' belongs here.

Thanks for catching this formatting error. We have removed this header.

Line 19: "COVID-19 is spread through respiratory droplets that can be transmitted during close, person-to-person interactions": Please add a reference which states that this is indeed the case for SARS-CoV-2, as all references here pre-date the pandemic.

We thank the Reviewer for pointing this out. We have added two additional references that state that this is the case for SARS-CoV-2: Wu and McGoogan, 2020 and Yuki, Fujiogi and Koutsogiannaki, 2020 (see page 3).

***Results**:*

Figure 1 (caption): Please define the abbreviation 'MRP' in the main text before using it.

Thanks for the suggestion. We have defined the abbreviation MRP in the main text before introducing it (see page 4).

Line 75: Is there a specific reason Washington and South-Dakota were chosen to focus on here?

We focus on Washington and South Dakota as an example of two states with relatively low and high rates of contact intensity, respectively. We have clarified this in the text on page 14. Thank you for the suggestion.

Figure 2: Would it be possible to think of another way to present this which would make it easier to see the differences between states (although arranging the plots in the shape of the map of the US is a fun idea)?

Figure 4: Same comment as Figure 2

We appreciate the suggestion for another way of presenting differences between states and acknowledge that presenting trends over time for many states is challenging. We have tried different approaches and have found these figures to be the most effective way to present these plots.

Line 119: Could you explain why you use relative R0 instead of calculating R0 outright - as this would be easier to interpret than using a relative R0 and then scaling it to the relative R0 of the youngest age group (which is a bit weird as well since this age group lacks sufficient data)?

We apologize for the confusion and we thank the author for this helpful clarification. We have dropped the confusing phrasing "relative R0" and instead refer to these estimates directly as each age group's contribution to the overall transmission. In the Supporting Information Section 2.5, we now calculate R0 outright using an age-stratified deterministic compartmental model (Davies et al. 2020).

Line 135: "Understanding why we observe these differential state-level age assortativity trends is an important topic for future research." : This statement may be better suited for the 'Discussion' section.

We thank the Reviewer for the suggestion. We agree and we have moved this statement to the discussion section.

Discussion:

Line 166: "Finally, the reported contacts by age could be smoothed within the modeling framework.": Could you explain further what is meant by this?

We thank the Reviewer for pointing out this description was insufficient. We have now clarified in the text that the smoothing refers to statistical methods for minimizing large jumps between estimated number of contacts in adjacent age groups (see page 21).

Methods:

I think it would be good to rewrite the Methods section for additional clarity (especially subsection 4.2): it would be better to be very clear about what is novel about what you are doing (i.e. the use of this technique in the context of contact matrices + the extensions you are adding to the technique), before going further into detail.

Thanks very much for this suggestion. We have restructured the paper so the "data and methods" section comes before the results and discussion; this helps foreground the methods section. We now begin the Methods sections with a high level overview of our method and contrast our method with past literature to be more clear about what is novel about our approach.

Line 187: "this matrix": what matrix?

We thank the Reviewer for pointing this out. We have clarified the matrix we were referring to (see page 6).

Line 282: 'C' is not defined (although it is easy to guess what it refers to).

We thank the Reviewer for the suggestion. We have added a new paragraph more explicitly defining the contact matrix ‘C’ (see page 5).

Line 345: Figure 1 does not show all states but only 2 of them.

Thanks for pointing this out. We have corrected this typo.

Supplemental Information:

'Guidance to Researchers': This section is not referred to in the text, and it is not very clear what the purpose of the section here is.

We thank the Reviewer for pointing this out. We have removed this section from the Supporting Information and distilled key aspects of this section into the discussion section of the paper (see page 20).

Line 686: “We calculate R0 assuming a baseline R0 value of 2.54 for pre-pandemic contact rates.”: Could you add a reference to justify the use of this number?

We thank the author for suggesting we provide a reference justifying this number. We chose this value based on past studies, which estimated the relative R0 for COVID-19 to be approximately 2.5. We now cite (Viceconte and Petrosillo, 2020) as justification (see Supporting Information, page 9).

Bibliography

(1) Davies, Nicholas G., Petra Klepac, Yang Liu, Kiesha Prem, Mark Jit, CMMID COVID-19 working group, Carl A. B. Pearson, Billy J. Quilty, Adam J. Kucharski, Hamish Gibbs, Samuel Clifford, Amy Gimma, Kevin van Zandvoort, James D. Munday, Charlie Diamond, W. John Edmunds, Rein M. G. J. Houben, Joel Hellewell, Timothy W. Russell, Sam Abbott, Sebastian Funk, Nikos I. Bosse, Yueqian Fiona Sun, Stefan Flasche, Alicia Rosello, Christopher I. Jarvis, and Rosalind M. Eggo. 2020. “Age-Dependent Effects in the Transmission and Control of COVID-19 Epidemics.” *Nature Medicine* 26(8):1205–11. doi: 10.1038/s41591-020-0962-9.

(2) Gelman, Andrew, Jeffrey Lax, Justin Phillips, Jonah Gabry, and Robert Trangucci. 2018. “Using Multilevel Regression and Poststratification to Estimate Dynamic Public Opinion.” 48.

(3) Viceconte, Giulio, and Nicola Petrosillo. 2020. “COVID-19 R0: Magic Number or Conundrum?” *Infectious Disease Reports* 12(1):8516. doi: 10.4081/idr.2020.8516.
