# Supplementary material for: Novel estimates reveal subnational heterogeneities in disease-relevant contact patterns in the United States
Source: PLoS Comput Biol. 2022 Dec 2;18(12):e1010742. doi: 10.1371/journal.pcbi.1010742 (PMC9749998; doi:10.1371/journal.pcbi.1010742)
Supplement: Attachment — Submitted filename: bics_mrp_response_letter_revision2_sep27_2022.pdf [file pcbi.1010742.s003.pdf]

Submission #PCOMPBIOL-D-22-00658 for PLOS Computation Biology
Response to Reviewers (Round 2)

Dear Dr. Daniel B Larremore and the Editorial Team,

Thank you for giving us the opportunity to submit a revised version of our manuscript, "Novel Estimates Reveal Subnational Heterogeneities in Disease-Relevant Contact Patterns in the United States" for publication in *PLOS Computational Biology*. We thank the reviewers again for their careful read of our revised manuscript and constructive advice for improvement. We have addressed the outstanding concern of Reviewer 1 as detailed below.

Response to specific comments from Reviewer 1:

Reviewer #1: I thank the authors for having addressed my comments, especially the additional sensitivity analysis. However I think that the inclusion of the Non-Pharmaceutical-Intervention (NPI) in the model (included in the additional material and never mentioned in the text) should be discussed in the main text. A vast majority of works [Gimma et al. 2020, Coletti et al. 2020, Liu et al. 2021, Latsubaia et al. 2020, Tizzani et al. 2022] have shown a very strong effect of NPIs on the number of contacts. As such, the result of NPIs reducing the performance of the model is quite surprising and warrants further discussion in the main paper. In such a way, the reader is aware that these estimates of contact patterns are against the trend measured in these studies.

We thank the reviewer for this suggestion. We have added a new paragraph into the results section on the addition of the Non-Pharmaceutical Intervention covariate (NPI) in the model. In this paragraph, we now also cite the reviewer's suggested literature on the effect of NPIs on the number of contacts **(page 20)**. We agree that understanding why incorporating NPIs into our model did not improve its predictive accuracy is a potential avenue for future research.

Response to specific comments from Reviewer 2:

Reviewer #2: This new version addresses all of my suggestions. The internal validation check in S3.1 and the short discussion of validation on page 21 are both very helpful.

We thank the reviewer very much for taking the time to review our paper. We appreciate their feedback and helpful suggestions for improvement.
